# Thermally Stable Donor–Acceptor Type (Alkynyl)Gold(III) TADF Emitters Achieved EQEs and Luminance of up to 23.4% and 70 300 cd m^−2^ in Vacuum‐Deposited OLEDs

**DOI:** 10.1002/advs.201802297

**Published:** 2019-08-06

**Authors:** Dongling Zhou, Wai‐Pong To, Yoonhyun Kwak, Yongsuk Cho, Gang Cheng, Glenna So Ming Tong, Chi‐Ming Che

**Affiliations:** ^1^ State Key Laboratory of Synthetic Chemistry Institute of Molecular Functional Materials Department of Chemistry The University of Hong Kong Pokfulam Road Hong Kong SAR China; ^2^ Organic Electronic Materials Lab. Samsung Advanced Institute of Technology 130 Samsung‐ro, Yeongtong‐gu Suwon‐si Gyeonggi‐do 16678 Republic of Korea; ^3^ HKU Shenzhen Institute of Research and Innovation Shenzhen Guangdong 518053 China; ^4^ Sichuan Knowledge Express Institute for Innovative Technologies World Financial Center Gaoxin District Chengdu 610041 China

**Keywords:** gold, ligand‐to‐ligand charge transfer, organic light‐emitting devices, thermally activated delayed fluorescence

## Abstract

Thermally stable, strongly luminescent gold‐TADF emitters are the clue to realize practical applications of gold metal in next generation display and lighting technology, a scarce example of which is herein described. A series of donor–acceptor type cyclometalated gold(III) alkynyl complexes with some of them displaying highly efficient thermally activated delayed fluorescence (TADF) with Φ up to 88% in thin films and emission lifetimes of ≈1–2 µs at room temperature are developed. The emission color of these complexes is readily tunable from green to red by varying the donor unit and cyclometalating ligand. Vacuum‐deposited organic light‐emitting diodes (OLEDs) with these complexes as emissive dopants achieve external quantum efficiencies (EQEs) and luminance of up to 23.4% and 70 300 cd m^−2^, respectively.

## 1 Introduction

Gold is a 3rd row transition metal with a large spin–orbit coupling constant and much higher earth abundance than iridium. However, contrary to iridium that has been extensively used in organic light‐emitting diode (OLED) technology, examples of gold OLED with practical interests are scarce. The first example of gold OLED was reported by Ma and Che in 1999^1^, ^2^ with inferior performance, and device performance has been improved in recent years exploiting luminescent cyclometalated Au(III) complexes.^3^, ^4^, ^5^, ^6^, ^7^ Due to the electrophilic nature/relatively high reduction potential of Au(III) ion, the luminescent excited states of Au(III) complexes are usually ligand centered with little metal character, resulting in long triplet emission lifetimes,^8^, ^9^, ^10^, ^11^, ^12^, ^13^, ^14^, ^15^ thereby posing significant challenges to achieve high performance OLEDs with low efficiency roll‐off at high luminance and driving voltage. A relatively short triplet emission lifetime is not only important to suppress efficiency roll‐off but also crucial to achieve stable device with long operation lifetimes.^16^, ^17^, ^18^, ^19^, ^20^ In this regard, thermally stable gold TADF emitters with high emission quantum yields are appealing. Recently, we and Linnolahti, Bochmann, Credgington and coworkers independently reported strongly luminescent Au(III) and Au(I) TADF emitters,^21^, ^22^, ^23^ respectively, and successfully use these emitters in the fabrication of high performance gold OLEDs with external quantum efficiencies (EQEs) up to 23.8% and 27.5%, and efficiency roll‐off down to 1% and 4%, respectively. Although the pincer Au(III)‐aryl TADF emitters with emission lifetime <2 µs are appealing emissive dopant for solution processed OLEDs,^21^ their use in vacuum‐deposited OLEDs is hampered by the thermal stability of gold‐aryl bond. Since Au(III)−C_sp_(acetylide) bond is stronger than Au(III)−C_sp2_(aryl) one, we conceive to develop stable Au(III)‐acetylide TADF emitters constructed with donor–acceptor units. The (C^^^N^^^C)‐Au(III) system bearing a deprotonated 2,6‐bis(2,4‐difluorophenyl)pyridine ligand would have higher triplet ligand‐centered excited state (^3^IL) and larger energy separation between ^3^IL/^3^LLCT (LLCT = ligand‐to‐ligand charge transfer) than that bearing an unsubstituted C^^^N^^^C ligand, which would be beneficial to achieve efficient TADF. In this work, we report a series of Au(III) acetylide emitters with emission color spanning from green to red and emission quantum yields of up to 60% in solutions and 88% in thin films. Emission quantum yield measurements conducted at different temperatures, together with density functional theory calculations, suggested that the gold(III) acetylide complexes with a strong donor moiety display TADF. Vacuum‐deposited OLEDs fabricated with these emitters showed high efficiency (EQE up to 23.4%) and small efficiency roll‐off (down to 5.6%) at 1000 cd m^−2^, which are comparable to the best reported Ir(III) and Pt(II) emitters.^24^, ^25^, ^26^, ^27^, ^28^

## 2 Results

### 2.1 Synthesis and Characterization

**Figure 1:**
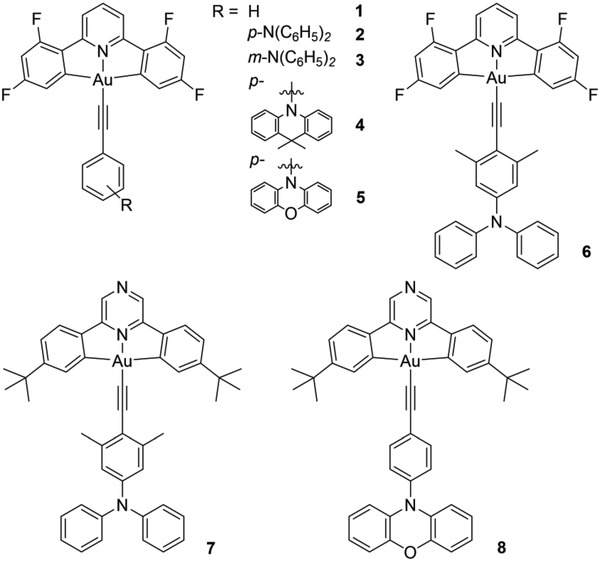
Chemical structures of complexes 1–8.

The Au(III) acetylide complexes reported herein are shown in **Figure** **1**. They were synthesized with moderate yields (45–68%) according to the reported methods as detailed in the Supporting Information. Except for 1, this series of Au(III) complexes was designed to have donor–acceptor frameworks for achieving TADF.^21^ Acetylide ligands with different amino‐substituents have been employed to tune the photophysical properties of these complexes. Complexes 1 and 3–5 have low solubility in organic solvents (e.g., dichloromethane, chloroform, DMSO), rendering it difficult to obtain their ^13^C NMR spectra. They are air‐stable and appear yellow in the solid state. Thermal stability of 1–8 was investigated using thermogravimetric analysis (TGA) method, with decomposition temperature *T*_d_ (defined as the temperature at which the complex shows a 2% weight loss) up to 347 °C under a nitrogen atmosphere (Figure S5, Supporting Information).

Single crystals of 5 were obtained by slow evaporation of a solution of the complex in fluorobenzene. The crystal structure of 5 is depicted in Figure S6 in the Supporting Information. The gold atom adopts a distorted square planar geometry with C–Au–C angle of [Au(C^^^N^^^C)] moiety and N–Au–C(acetylide) angle being 163.4° and 177.9°, respectively. The C^^^N^^^C ligand makes a dihedral angle of 47.6° with the phenyl ring of the acetylide ligand. There are intermolecular π–π stacking interactions between [C^^^N^^^C] ligands with interplanar distance of ≈3.4 Å.

### 2.2 Photophysical Properties

**Figure 2:**
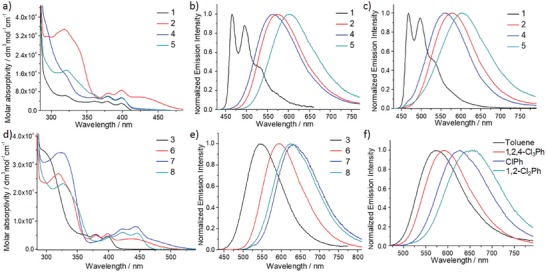
a) Absorption spectra of 1, 2, 4, and 5 in toluene, b) emission spectra of 1, 2, 4, and 5 in degassed toluene at room temperature, c) emission spectra of 1, 2, 4, and 5 in PMMA thin films (4 wt%), d) absorption spectra of 3, 6, 7, and 8 in toluene, e) emission spectra of 3, 6, 7, and 8 in degassed toluene at room temperature, and f) emission spectra of 2 in different solvents at room temperature.

The UV–vis absorption and emission spectral data of 1–8 are summarized in **Table** **1**. The absorption and emission spectra of 1–8 are depicted in **Figure** **2**. In toluene, complexes 1–6 exhibit intense absorption bands at λ = 290–340 nm (ε = 7 × 10^3^–4 × 10^4^ dm^3^ mol^−1^ cm^−1^), and moderately intense, vibronic‐structured absorption bands at λ = 360–400 nm [ε = (3–9) × 10^3^ dm^3^ mol^−1^ cm^−1^] at room temperature. Due to the stabilized lowest unoccupied molecular orbital (LUMO) of the 2,6‐bis(4‐(*tert*‐butyl)phenyl)pyrazine, the absorption bands of 7 and 8 are red‐shifted relative to those bearing di‐deprotonated 2,6‐bis(2,4‐difluorophenyl)pyridine. For 2–8, obvious absorption tail extending to 525 nm is observed, especially for the complexes containing diphenylamine substituent. The low‐energy structured absorption bands and absorption tail are assigned to metal‐perturbed intraligand π–π* transitions (^1^IL) of tridentate [C^^^N^^^C] ligand and ligand‐to‐ligand charge transfer transitions (^1^LLCT) from π (amine‐based substituent) to π* (C^^^N^^^C ligand), respectively.^3^, ^21^

**Table 1:** Photophysical data of Au(III) complexes at room temperature

	Absorption	Emission	
	λ_abs_ [nm] (ε [×10^3^ mol^−1^ dm^3^ cm^−1^])^a)^	In toluene	In 4 wt% in thin film
		λ_em_ [nm] (Φ; τ [µs]; *k*_r_ [10^5^ s^−1^])^b)^	λ_em_ [nm] (Φ; τ [µs]; *k*_r_ [10^5^ s^−1^])
1	319(6.52), 359(4.01), 378(3.97), 398(3.13)	466, 495, 530 (0.002; 0.34; 0.06)	468, 496, 530^c)^ (0.028; 22.4; 0.01)
2	294(31.78), 318(34.69), 379(7.39), 398(8.66), 426(br, 5.68)	574 (0.60; 0.78; 7.69)	577^c)^ (0.88; 0.85; 10.35)
3	295(34.90), 359(5.35), 379(5.43), 398(4.72)	545 (0.21; 1.25; 1.68)	546^c)^ (0.29; 3.78; 0.77)
4	320(15.24), 359(5.12), 379(6.17), 398(5.53)	562 (0.49; 0.80; 6.13)	560^c)^ (0.67; 1.43; 4.69)
5	290(20.45), 321(17.27), 360(6.77), 379(5.90), 399(5.28)	603 (0.57; 0.84; 6.79)	567^d)^ (0.65; 1.46; 4.45)
6	319(26.74), 338(20.49), 380(4.99), 398(5.59), 435(br, 3.78)	594 (0.25; 0.33; 7.58)	568^d)^ (0.80; 1.19; 6.72)
7	323(34.09), 398(4.63), 422(7.26), 443(8.15), 480(br, 2.88)	632 (0.02; 0.20; 1.00)	595^e)^ (0.56; 0.77; 7.27)
8	327(23.16), 400(4.31), 423(6.09), 445(5.75)	625 (0.08; 0.25; 3.20)	527, 570, 640^c)^ (0.09; 0.33; 2.73)^f)^

^a)^ In toluene at 2 × 10^−5^ mol dm^−3^. “br” stands for broad

^b)^ Emission quantum yields (Φ) were measured with 9,10‐bis(phenylethynyl)‐anthracene in benzene as the standard (Φ = 0.85)

^c)^ PMMA thin film samples (with 4 wt% of Au(III) complex)

^d)^ TCTA:TPBi (1:1) thin film samples (with 4 wt% of Au(III) complex)

^e)^ MCP thin film samples (with 4 wt% of Au(III) complex)

^f)^ Emission lifetime of 8 was measured at 640 nm.

**Figure 3:**
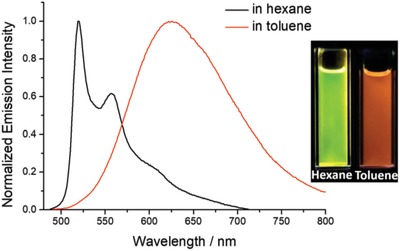
Emission spectra and photograph of 8 (concentration = 2 × 10^−5^ mol dm^−3^) in degassed hexane and toluene.

Upon photoexcitation, complexes 1–8 display green to red emission in degassed toluene. The emission of 1 features vibronic structures with low emission quantum yield of 0.2% and is insensitive to solvent polarity. Together with the large Stokes shift (6400 cm^−1^) and small radiative decay rate constant (*k*_r_) (6 × 10^3^ s^−1^), the emission of 1 is assigned as phosphorescence originating from metal‐perturbed intraligand π to π* transition of the C^^^N^^^C ligand.^8^ Complexes 2–8 with amino‐substituted acetylide exhibit broad emission band with high emission quantum yields (up to 60%) and more than 100‐fold faster *k*_r_ (of the order 10^5^ s^−1^) than complex 1. The emission of 2 shows positive solvatochromism with the emission maximum red‐shifted by 80 nm (2130 cm^−1^) upon changing the solvent from toluene to *o*‐dichlorobenzene (Figure 2f). The Lippert–Mataga plot with a positive slope of 1.1 × 10^4^ cm^−1^ suggests a much larger dipole moment in the emissive excited state than the ground state. Therefore, the emissive excited state of 2 is suggested to have significant charge‐transfer character. On the other hand, the emission energy and profile of 8 shows significant dependence on solvent polarity. As shown in **Figure** **3**, there is not only a blue‐shift in emission maximum upon changing the solvent from toluene to nonpolar hexane, but also a drastic change in emission profile from structureless, broad band in toluene to vibronically structured bands in hexane, with a concomitant decrease in *k*_r_ from 3.24 × 10^5^ s^−1^ (in toluene) to 6.21 × 10^3^ s^−1^ (in hexane). Since the observed vibronic spacings of 1300–1400 cm^−1^ and *k*_r_ in the order of 10^3^ s^−1^ are typical traits of ^3^IL emission of gold(III) complexes supported by C^^^N^^^C ligands, the emission of 8 in hexane is assigned as the ^3^IL_C^N^C_ emission.^9^, ^12^, ^29^, ^30^ When switching to nonpolar solvent, the charge‐transfer excited state may be destabilized and becomes higher lying than ^3^IL excited state, thus resulting in the emission being mainly derived from ^3^IL excited state in nonpolar hexane.

Based on our previous work on gold(III) aryl complexes,^21^ the much faster *k*_r_ of the order 10^5^ s^−1^ may suggest TADF to be operative in the emission mechanism of the gold(III) acetylide complexes 2–8 investigated herein. To gain further insight into the emission origin, the emission properties of 1–8 were also measured at 60 °C in degassed toluene. The temperature‐dependent emission spectra and data are given in the supporting information (Figure S2 and Table S2, Supporting Information). In principle, if the emission origin is phosphorescence from a single conformation, the radiative decay rate constant, *k*_r_^P^, should be more or less temperature‐independent; on the other hand, if the emission mechanism involves TADF, *k*_r_ should be temperature‐dependent and increases with temperature. For complexes 2–6, their *k*_r_ were found to increase (by 19–42%) with temperature, indicating that TADF is a plausible emission mechanism for these complexes. However, since the aryl moiety of the acetylide ligand is free to rotate, these complexes may also adopt multiple conformations with different *k*_r_ and thus leading to temperature‐dependent *k*_r_. This would be addressed later in the computational section. For complexes 1 and 7, their nonradiative decay rates are too fast that a precise measurement of the emission lifetime cannot be obtained. The *k*_r_ of complex 8 was found to decrease with temperature.

**Figure 4:**
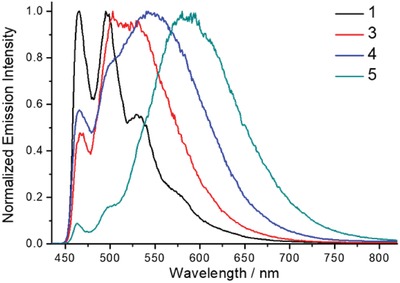
Emission spectra of 1 and 3–5 (4 wt%) in PMMA thin films at 77 K.

The emission spectra of 1–8 in poly(methyl methacrylate) (PMMA) thin films were also examined and that of 1, 2, 4, and 5 are shown in Figure 2c and that of 3, 6–8 are shown in Figure S1 in the Supporting Information. The emission profiles are similar to those in solutions with emission quantum yields of up to 88% and *k*_r_ values of (0.01–10.35) × 10^5^ s^−1^. The effect of temperature on the emission of 2–5 in PMMA thin films was examined. For complexes 2 (Figure S4, Supporting Information) and 5, their structureless, broad emission profiles in PMMA thin films remain largely unchanged upon cooling from room temperature to 77 K except for 5 which shows relatively weak vibronic‐structured bands (λ_em_ at 460–500 nm) at 77 K (**Figure** **4**). Contrary to 2 and 5, there are obvious changes in the emission profiles for 3 and 4 at 77 K, with obvious vibronic‐structured emission bands emerging at λ_em_ = 460–500 nm. By comparing the emission spectra of 1 and 3–5 in PMMA thin films at 77 K (Figure 4), the vibronically structured emission bands of 3–5 in PMMA thin films (460–500 nm) were found to be identical in energy to that of 1 observed under the same conditions. The vibronic‐structured emission bands (460–500 nm) of 3–5 are thus assigned as the phosphorescence localized on the C^^^N^^^C ligand. Because the emission profiles of 3 and 4 in PMMA thin films show high sensitivity to temperature, variable‐temperature emission of 3 and 4 in thin films was investigated. For 4, high‐energy, vibronically structured emission band gradually develops upon decreasing the temperature from 298 to 77 K (Figure S3, Supporting Information). The emission at 465 nm displays monoexponential decay with time constant τ_465_ = 105–179 µs in the temperature range 77–227 K; on the other hand, the emission at 498 nm can be fitted into two exponentials with a short‐lived component τ_498,s_ = 7.3–61.6 µs and a long‐lived component τ_498,l_ = 107–182 µs at 77–227 K, with the contribution of the long‐lived component increasing upon lowering the temperature (Figure S3b, Supporting Information). The long‐lived component τ_498,l_ and τ_465_ have similar time constants over the temperature range 77–227 K, suggesting that they have the same emission origin. Since the emission at 465 nm is assigned to metal‐perturbed intraligand π to π* transition (^3^IL) of the C^^^N^^^C ligand, the long‐lived component at 498 nm can also be attributed to the ^3^IL(C^^^N^^^C) transition. The contribution of the short‐lived component τ_498,s_ decreases with decreasing temperature, indicating that the corresponding emitting state is thermally populated. Since TADF is also a thermally activated process, this short‐lived component is tentatively assigned as being derived from TADF.

### 2.3 Electrochemistry

To gain further insight into the relationship between structures and emission, their electrochemical properties were investigated by cyclic voltammetry in *N*,*N*‐dimethylformamide with 0.1 mol dm^−3^ [*n*Bu_4_N]PF_6_ as supporting electrolyte. The electrochemical data are summarized in Table S4 in the Supporting Information. Complexes 2–8 show one irreversible oxidation wave with *E*_pa_ at 0.78–1.07 V, one reversible reduction couple at −1.06 to −1.22 V and one irreversible reduction wave at −1.81 to −1.90 V versus SCE. Complex 1 displays reduction peaks but oxidation is not observed within the solvent window. Complexes 1–6 with the same C^^^N^^^C ligand show reduction waves at similar potentials. Except for 5 and 8 with a phenoxazine on the acetylide donor ligand, other Au(III) complexes show irreversible oxidation wave. The oxidation waves are assigned to the oxidation of the amino‐substituted acetylide, while the first reduction couples are attributed to the reduction of C^^^N^^^C/C^^^N^PZ^^^^C ligands.

According to the electrochemical data, phenylacetylide with a diphenylamine at *para* position is more electron‐rich than that having the same group at *meta* position (0.99 V in comparison with 1.07 V), leading to a red shift of emission in toluene for the former complex (574 nm for 2; 545 nm for 3). Complexes having phenoxazine (5), diphenylamine (2) and 9,9‐dimethyl‐9,10‐dihydroacridine (4) on the phenylacetylide ligand show decreasing highest occupied molecular orbital (HOMO) with oxidation potential at 0.82, 0.99, and 1.03 V respectively, which correlates with the trend in their emission energy where the complex with a higher‐lying HOMO has lower emission energy (603 nm for 5; 574 nm for 2; 562 nm for 4).

### 2.4 Computational Study

**Figure 5:**
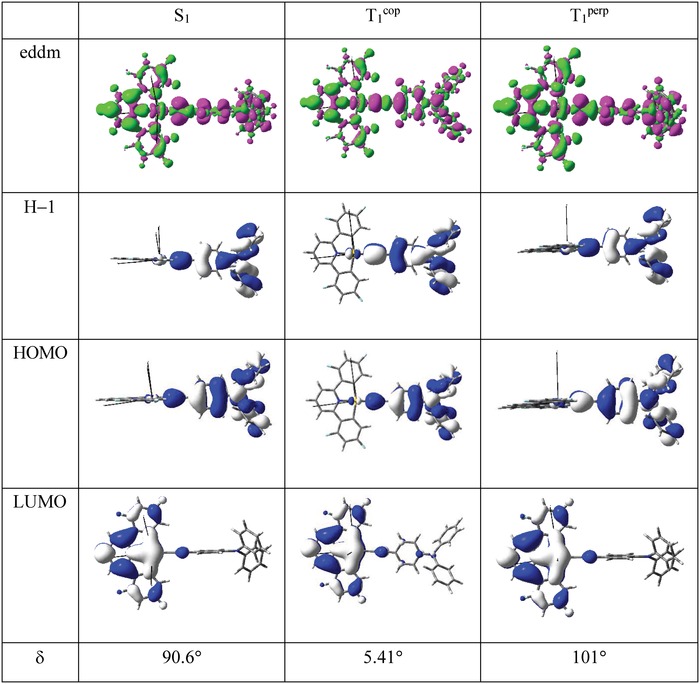
Electron density difference map (eddm), molecular orbital (MO) surfaces of the optimized S_1_ and T_1_ excited states with δ at their respective optimized geometries. Color code for the eddm: green, increase in electron density; magenta, decrease in electron density.

The typical triplet radiative decay rates for Au(III) complexes are usually of the order 10^3^ s^−1^ or less. Given that their emissive excited states are mostly ligand‐centered in nature, the unusually fast *k*_r_ of the order 10^5^ s^−1^ for 2–8 thus suggests emissive origins different from ^3^IL. DFT/TDDFT calculations were performed with 2 as a representative example. Both the optimized lowest singlet (S_1_) and triplet excited states (T_1_) are mainly derived from a HOMO → LUMO transition, with the T_1_ excited state having an additional minor contribution (<2%) from the H‐1 → LUMO transition. The H‐1, HOMO, and LUMO surfaces are depicted in **Figure** **5**. Both the H‐1 and HOMO are localized mostly on the TPA motif (TPA stands for triphenylamine) with little Au character (<5%), while the LUMO is mainly localized on the C^^^N^^^C ligand; therefore the S_1_ and T_1_ excited states are best described as ^1,3^LLCT excited states, respectively. The optimized ^3^IL(C^^^N^^^C) excited state is found to lie more than 2200 cm^−1^ above the ^3^LLCT excited state. Since the presence of a C≡C bond increases the distance between the phenyl moiety of arylacetylide and the C^^^N^^^C ligand framework, the steric hindrance between the protons on the phenyl moiety of arylacetylide and those on the phenyl moiety of C^^^N^^^C ligand will be small and this may allow rotational flexibility of the TPA motif. Two local minima were found for the T_1_ excited state: one at δ = 5.4° (T_1_^cop^) and the other at δ = 101° (T_1_^perp^), with the former more stable by ≈200–300 cm^−1^ (δ is the dihedral angle between the C^^^N^^^C plane and the phenyl ring attached to the C≡C bond). As there are many vibrational modes smaller than 200 cm^−1^ (Figure S10, Supporting Information), there should be a distribution of T_1_ excited state with varied δ at room temperature. The corresponding S_1_–T_1_ energy gap (Δ*E*_ST_) decreases from 2660 cm^−1^ at the T_1_^cop^ optimized geometry to 182 cm^−1^ at the T_1_^perp^ optimized geometry due to the concomitant decrease in HOMO–LUMO overlap (Table S9, Supporting Information).

The computed *k*_r_ of T_1_ phosphorescence and S_1_ prompt fluorescence at the optimized T_1_ geometries are presented in **Table** **2**. The *k*_r_ of T_1_ phosphorescence ranges from 2.70 × 10^2^ s^−1^ at δ = 5.4° to 1.43 × 10^3^ s^−1^ at δ = 101°; thus, pure phosphorescence cannot account for the experimental *k*_r_ values of 10^5^–10^6^ s^−1^, even after taking into account the different conformations that exist at room temperature. If the TADF mechanism is included to calculate the average *k*_r_ (Equation S1 in the Supporting Information),^31^ the *k*_r_ computed would be 5.13 × 10^2^ s^−1^ at δ = 5.4° and 1.23 × 10^6^ s^−1^ at δ = 101°. Taking into consideration that the *k*_r_ estimated from experimental data are a sum of all the radiative decay channels at all the thermally accessible dihedral angles, the average *k*_r_ would be of the order of 10^5^ s^−1^, which is of the same order of magnitude as that experimentally determined in toluene solution at room temperature (Further details could be found in the Supporting Information). Hence, TADF is the emission mechanism most consistent with the experimental results. It should be noted that at the optimized S_1_ excited state, the computed Δ*E*_ST_ is down to 18 cm^−1^ due to the near orthogonal disposition of the donor (TPA) and the acceptor (C^^^N^^^C) moieties (δ = 90.6°). In this situation, TADF is thus envisioned to be facile at the optimized S_1_ geometry. However, due to the orthogonal disposition of the D–A moieties, the oscillator strength (*f*) is ≈0 and the radiative decay rate at this geometry is much smaller (*k*_r_^F^(S_1_) < 10^3^ s^−1^). Thus, a small Δ*E*_ST_ is beneficial for facile TADF but it does not guarantee a large *k*_r_ (Table S10, Supporting Information ).

**Table 2:** Radiative decay rate constants at different optimized excited state geometries

	λ_em_ [nm]	*k*_r‐phosphorescence_ [s^−1^]	*k*_r‐fluorescence_ [s^−1^]	*k*_r,avg_/s^−1^
T_1_^cop^	561	2.70 × 10^2^	4.34 × 10^8^	5.13 × 10^2^
T_1_^perp^	570	1.43 × 10^3^	1.04 × 10^7^	1.23 × 10^6^

### 2.5 Electroluminescence Properties

**Figure 6:**
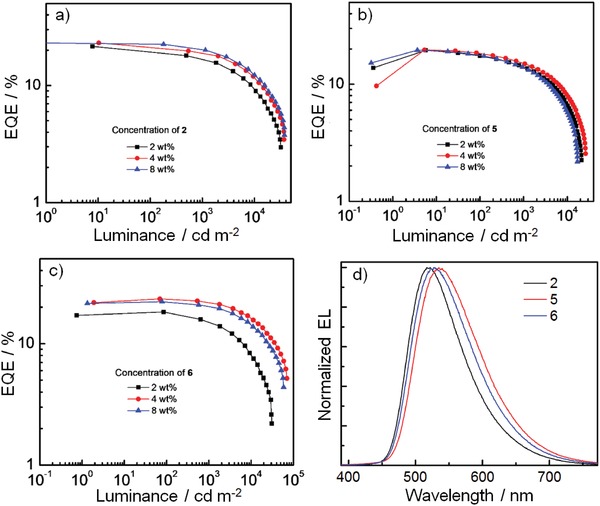
a–c) EQE–luminance characteristics of OLEDs based on 2, 5, and 6 with dopant concentration of 2, 4, and 8 wt%, and d) normalized EL spectra of devices with dopant concentration of 4 wt%.

To investigate the electroluminescent (EL) properties of these Au(III) complexes, vacuum‐deposited OLEDs based on 2, 5, and 6 were fabricated and characterized. The devices had a structure of ITO/HAT‐CN(5 nm)/TAPC (40 nm)/TCTA(10 nm)/TCTA:TPBi:Au(III) emitter(10)/TPBi(10 nm)/TmPyPb (40 nm)/LiF (1.2 nm)/Al (100 nm), in which HAT‐CN (1,4,5,8,9,11‐hexaazatriphenylene hexacarbonitrile) was used as a hole‐injecting layer, TAPC (di‐[4‐(*N*,*N*‐ditolyl‐amino)‐phenyl]cyclohexane) as a hole‐transporting layer and TmPyPb (1,3,5‐tri(m‐pyrid‐3‐yl‐phenyl)benzene) as an electron‐transporting layer. The emitting layer (EML) was constructed by doping 2, 5, or 6 in the co‐host system consisting of TCTA:(4,4′,4′′‐tris(carbazol‐9‐yl)‐triphenylamine) and TPBi [2,2′,2′′‐(1,3,5‐benzinetriyl)‐tris(1‐phenyl‐1‐H‐benzimidazole)] with 1:1 weight ratio. Two 10‐nm‐thick layers of TCTA and TPBi were inserted between EML and charge‐transporting layers as exciton‐blocking layers. The dopant concentrations of Au(III) emitters were 2, 4, and 8 wt%. Normalized EL spectra of OLEDs based on 2, 5, or 6 with 4 wt% dopant concentration were depicted in **Figure** **6**. The EL spectra of 2, 5, and 6 were respectively blue‐shifted by 46, 27, and 31 nm when compared with their PL in TCTA:TPBi thin film (Figure S7, Supporting Information). As aforementioned, the emission energy of donor–acceptor type Au(III) complexes studied in this work is sensitive to the polarity of the surrounding environment. The blue‐shift in EL spectra of 2, 5, and 6 could be the result of electronic field applied upon the thin EML.

EQE–luminance characteristics of the OLEDs based on 2, 5, and 6 with concentrations of 2, 4, and 8 wt% are depicted in Figure 6, and the corresponding key parameters are summarized in **Table** **3**. High EQE_max_ values of ≈23% were achieved in both 2 and 6 devices while relatively lower EQE_max_ of 19.7% in 5 device. The EQE values of devices fabricated with 2 (4 and 8 wt%) and 6 (4 and 8 wt%) are slightly higher than those of gold(III)‐OLEDs reported recently.^32^ In 4 wt% TCTA:TPBi (1:1) thin films, the PLQYs of 2 (0.86) and 6 (0.84) are higher than that of 5 (0.73), accounting for the high EQE_max_ values of 2 and 6 devices. Meanwhile, the shorter τ (1.06 µs) of 6 could explain the relatively smaller efficiency roll‐off of 5.6% at 1000 cd m^−2^ and high luminance of beyond 70 000 cd m^−2^ for the 6 device. Thanks to a low turn‐on voltage of 2.4 V, high power efficiency of 99.4 lm W^−2^ was achieved in the 2 device at the dopant concentration of 8 wt%. To the best of our knowledge, this is the highest value among Au‐OLEDs.^3^, ^4^, ^5^, ^6^, ^7^, ^21^, ^22^, ^32^ The device performance and operational lifetime of OLEDs based on 6 have been evaluated by Samsung with industry standard (see the Supporting Information for details). The OLED with 6 (5 wt%) exhibited an EQE of 17% at a current efficiency of 53 cd A^−1^ and a small efficiency roll‐off of 6% was observed. Its LT_95_ (an operational lifetime to 95% of initial luminance) at an initial luminance of ≈8000 cd m^−2^ was measured to be approximately 0.3 h. This corresponds to about 10 h at a practical luminance of 1000 cd m^−2^ and about 500 h at a luminance of 100 cd m^−2^. The estimated LT_95_ of this unoptimized device are comparable to those of recently reported gold(III)‐OLEDs (Table S7, Supporting Information).^32^

**Table 3:** Key performances of OLEDs based on 2, 5, and 6 as emitters

Complex	Dopant Concentration [wt%]	Maximum Luminance [cd m^−2^]	Maximum Current Efficiency [cd A^−1^]	Maximum Power Efficiency [lm W^−1^]	EQE [%]		CIE^a)^ (*x*, *y*)
					Maximum	At 1000 cd m^−2^	
2	2	32 000	67.0	81.0	21.6	17.0	0.32, 0.56
	4	37 000	71.9	87.0	23.1	19.2	0.34, 0.56
	8	37 500	76.0	99.4	23.1	20.1	0.35, 0.56
5	2	21 300	59.9	72.3	19.5	14.0	0.38, 0.56
	4	26 300	57.5	86.9	19.7	15.0	0.41, 0.55
	8	17 200	53.8	65.0	19.5	14.0	0.43, 0.53
6	2	30 900	55.5	69.5	18.2	14.9	0.33, 0.56
	4	70 300	70.6	82.8	23.4	22.1	0.40, 0.55
	8	59 100	66.3	79.6	22.2	20.4	0.41, 0.54

^a)^ Commission internationale de l'éclairage (CIE) coordinates at 1000 cd m^−2^.

## 3 Conclusion

In summary, we have presented a series of donor–acceptor type cyclometalated Au(III) alkynyl complexes exhibiting highly efficient photoluminescence with quantum yields of up to 60% in solutions and 88% in thin films. The spatially separated donor and acceptor moieties, together with the use of cyclometalating ligands having suitably high ^3^IL excited state energy, allow the excited state of these complexes to decay efficiently via TADF mechanism as substantiated by DFT/TDDFT calculations. Vacuum‐deposited OLEDs fabricated with these thermally stable Au(III) TADF emitters show outstanding performance with maximum EQE of 22.1% at a luminance of 1000 cd m^−2^. These findings affirm the excellence of Au(III) TADF emitters as functional materials in real‐world applications.

## 4 Experimental Section

All chemicals, unless otherwise noted, were purchased from commercial sources and used without further purification. All solvents for reaction and photophysical studies were of HPLC grade, except for toluene which was purified by distillation before use for photophysical studies. Synthesis and characterization of ligands and precursor Au(III) complexes, the details of computational studies, and additional data on photophysical properties of Au(III) complexes and OLEDs are provided in the Supporting Information.

*Physical Measurements and Instrumentation*: NMR spectra were recorded on DPX 400 and 500 Bruker FT‐NMR spectrometer with chemical shift (in ppm) relative to nondeuterated solvent residual. Unless otherwise stated, all NMR spectra were recorded at room temperature. Mass spectra (MALDI and EI) were recorded on a Bruker ultrafleXtreme MALDI‐TOF/TOF mass spectrometer and a DFS high resolution magnetic sector mass spectrometer. Elemental analyses were performed at the Institute of Chemistry of the Chinese Academy of Sciences, Beijing. All absorption spectra were recorded on a Hewlett‐Packard 8453 diode array spectrophotometer. Steady‐state emission spectra were recorded on a Horiba Fluorolog‐3 spectrophotometer. Solutions for photophysical studies were degassed by using a high vacuum line in a two‐compartment cell with five freeze‐pump‐thaw cycles. Low temperature (77 K) emission spectra for glassy state and solid state samples were recorded in quartz tubes (4 mm internal diameter) placed in a liquid nitrogen Dewar flask with quartz windows. The emission quantum yield was measured with 9,10‐bis(phenylethynyl)anthracene (Φ = 0.85) in benzene as reference and calculated by: Φ_s_ = Φ_r_(*B*_r_/*B*_s_)(*n*_s_/*n*_r_)^2^(*D*_s_/*D*_r_), in which the subscripts s and r refer to sample and reference standard solution, respectively, *n* is the refractive index of the solvents, *D* is the integrated emission intensity and Φ is the luminescence quantum yield. The excitation intensity *B* is calculated by: *B* = 1–10^−^ *^AL^*, where *A* is the absorbance at the excitation wavelength and *L* is the optical path length (*L* = 1 cm in all cases). The refractive indices of the solvents at room temperature were taken from standard sources. Emission quantum yields of thin film samples were measured with Hamamatsu C11347 Quantaurus‐QY Absolute PL quantum yields measurement system. The thin films were prepared by drop‐cast from a chlorobenzene solution containing the Au(III) complex with PMMA or MCP or TCTA/TPBi (1:1). The solvent was evaporated at 80 °C and translucent films were obtained. The emission lifetime measurements were performed on a Quanta Ray GCR 150‐10 pulsed Nd:YAG laser system. Errors for λ values (±1 nm), τ (±10%), and Φ (±10%) are estimated.

*Crystal Structure Determination*: X‐ray diffraction data of the single crystals of 5 were collected on Bruker D8 VENTURE MetalJet Photon II CPAD X‐Ray Diffractometer. The diffraction images were interpreted and the diffraction intensities were integrated by using the program SAINT V8.34A (Bruker AXS Inc., 2013). The crystal structure was solved by Patterson method employing SHELXT 2014/5 (Sheldrick, 2014) and refined by using program SHELXL2016/6 (Sheldrick, 2016)/shelXle (C.B. Huebschle, rev 717).^33^ The crystallographic data are compiled in Table S6 in the Supporting Information. The crystal structure of 5 is depicted in Figure S6 in the Supporting Information.

*OLED Fabrication*: OLEDs were fabricated in a Kurt J. Lesker SPECTROS vacuum deposition system with a base pressure of 10^−8^ mbar. In the vacuum chamber, organic materials were thermally deposited in sequence at a rate of ≈0.1 nm s^−1^. The doping process in the emitting layer was realized by codeposition technology. Afterwards, LiF (1.2 nm) and Al (100 nm) were thermally deposited at rates of 0.03 and 0.2 nm s^−1^, respectively. Film thicknesses were determined in situ by calibrated oscillating quartz‐crystal sensors. EL spectra, *J*–*L*–*V* characteristices, CIE coordinates, EQE, CE, and PE were measured using a Keithley 2400 source‐meter and an absolute external quantum efficiency measurement system (C9920‐12, Hamamatsu Photonics). All devices were characterized at room temperature without encapsulation.

*Synthesis and Characterization of (C^^^N^^^C)‐Au Acetylides 1–8*: Complexes 1–8 were prepared from the reactions between (C^^^N^^^C)‐AuCl and the corresponding arylacetylenes in the presence of triethylamine and a catalytic amount of CuI in dichloromethane under N_2_ according to the literature method of preparing other (C^^^N^^^C)‐Au acetylides.^34^ The pure products were obtained by recrystallization with dichloromethane/methanol for three times.

1: 0.06 g of (C^^^N^^^C)‐AuCl used. Yield: 0.03 g (45%). ^1^H NMR (500 MHz, CD_2_Cl_2_, δ): 7.97 (t, *J =* 8.0 Hz, 1H), 7.88 (d, *J =* 8.0 Hz, 2H), 7.61 (dd, *J =* 6.5, 2.5 Hz, 2H), 7.57 (dd, *J =* 7.0, 1.5 Hz, 2H), 7.38–7.31 (m, 3H), 6.75–6.72 (m, 2H); ^19^F NMR (470 MHz, CD_2_Cl_2_, δ): −104, −108; IR (KBr): ν = 2159 cm^−1^ (*v*(C≡C)); EI‐MS: *m/z* 599.0571 [M]^+^. Anal. calcd for C_25_H_12_AuF_4_N: C 50.10, H 2.02, N 2.34; found: C 49.66, H 2.02, N 2.34.

2: 0.15 g of (C^^^N^^^C)‐AuCl used. Yield: 0.13 g (62%). ^1^H NMR (500 MHz, CD_2_Cl_2_, δ): 7.89 (t, *J* = 8.5 Hz, 1H), 7.79 (d, *J* = 8.0 Hz, 2H), 7.45(d, *J* = 6.0 Hz, 2H), 7.39 (d, *J* = 8.5 Hz, 2H), 7.29 (t, *J* = 7.5 Hz, 4H), 7.12 (d, *J* = 7.5 Hz, 4H), 7.06 (t, *J* = 7.5 Hz, 2H), 7.01 (d, *J* = 8.5 Hz, 2H), 6.74–6.68 (m, 2H); ^13^C NMR (126 MHz, CD_2_Cl_2_, δ): 168.04, 165.16 (dd, *J* = 261.6, 11.3 Hz), 162.09 (dd, *J* = 267.9, 10.1 Hz), 161.57, 161.52, 147.87, 147.52, 143.77, 133.03, 129.72, 125.07, 123.63, 123.18, 121.25 (d, *J* = 17.6 Hz), 119.67, 119.28 (d, *J* = 17.6 Hz), 103.43 (t, *J* = 26.4 Hz), 100.33, 89.58; ^19^F NMR (470 MHz, CD_2_Cl_2_, δ): −104, −108; IR(KBr): ν = 2153 cm^−1^ (*v*(C≡C)); MALDI‐MS: *m/z* 766.1391 [M]^+^. Anal. calcd for C_37_H_21_AuF_4_N_2_: C 57.98, H 2.76, N 3.66; found: C 57.68, H 2.82, N 3.62.

3: 0.06 g of (C^^^N^^^C)‐AuCl used. Yield: 0.05 g (60%). ^1^H NMR (500 MHz, CD_2_Cl_2_, δ): 7.91 (t, *J =* 8.0 Hz, 1H), 7.83 (d, *J =* 8.0 Hz, 2H), 7.48 (dd, *J =* 7.0, 2.5 Hz, 2H), 7.30–7.27 (m, 4H), 7.24–7.19 (m, 3H), 7.12 (dd, *J* = 8.5, 1.0 Hz, 4H), 7.06–7.00 (m, 3H), 6.71–6.67 (m, 2H); ^19^F NMR (470 MHz, CD_2_Cl_2_, δ): −104, −108; IR(KBr): ν = 2159 cm^−1^ (*v*(C≡C)); MALDI‐MS: *m/z* 766.1264 [M]^+^. Anal. calcd for C_37_H_21_AuF_4_N_2_·MeOH: C 57.15, H 3.16, N 3.51; found: C 56.74, H 2.79, N 3.46.

4: 0.10 g of (C^^^N^^^C)‐AuCl used. Yield: 0.08 g (55%).^1^H NMR (500 MHz, CDCl_3_, δ): δ 7.95 (t, *J* = 8.0 Hz, 1H), 7.86 (d, *J* = 8.0 Hz, 2H), 7.82(d, *J* = 8.0 Hz, 2H), 7.63 (dd, *J* = 6.5, 2.5 Hz, 2H), 7.47 (dd, *J* = 8.0, 1.5 Hz, 2H), 7.33 (d, *J* = 8.5 Hz, 2H), 7.01 (td, *J* = 7.5, 1.5 Hz, 2H), 6.94 (td, *J* = 8.0, 1.5 Hz, 2H,), 6.72–6.67 (m, 2H), 6.37 (dd, *J* = 8.0, 1.0 Hz, 2H), 1.70 (s, 6H); ^19^F NMR (470 MHz, CDCl_3_, δ): −103, −108; IR(KBr): ν = 2155 cm^−1^ (*v*(C≡C)); MALDI‐MS: *m/z* 791.1354 [M‐CH_3_]^+^. Anal. calcd for C_40_H_25_AuF_4_N_2_: C 59.56, H 3.12, N 3.47; found: C 59.19, H 2.99, N 3.36.

5: 0.12 g of (C^^^N^^^C)‐AuCl used. Yield: 0.09 g (53%). ^1^H NMR (500 MHz, CD_2_Cl_2_, δ): 8.00 (t, *J* = 8.0 Hz, 1H), 7.90 (d, *J* = 8.0 Hz, 2H), 7.79(d, *J* = 8.0 Hz, 2H), 7.64 (d, *J* = 6.0 Hz, 2H), 7.33 (d, *J* = 8.5 Hz, 2H), 6.76 (t, *J* = 10.5 Hz, 2H), 6.69–6.61 (m, 6H), 6.01 (d, *J* = 7.0 Hz, 2H); ^19^F NMR (470 MHz, CD_2_Cl_2_, δ): −104, −108; IR(KBr): ν = 2155 cm^−1^ (*v*(C≡C)); MALDI‐MS: *m/z* 780.1027 [M]^+^. Anal. calcd for C_37_H_19_AuF_4_N_2_O: C 56.94, H 2.45, N 3.59; found: C 56.63, H 2.19, N 3.52.

6: 0.20 g of (C^^^N^^^C)‐AuCl used. Yield: 0.18 g (60%). ^1^H NMR (500 MHz, CD_2_Cl_2_, δ): 7.97 (t, *J* = 8.0 Hz, 1H), 7.89 (d, *J* = 8.5 Hz, 2H), 7.68 (dd, *J* = 6.5, 2.0 Hz, 2H), 7.27 (t, *J* = 7.5 Hz, 4H), 7.09 (d, *J* = 8.0 Hz, 4H), 7.03 (t, *J* = 7.5 Hz, 2H), 6.81 (s, 2H), 6.75–6.70 (m, 2H), 2.49 (s, 6H); ^13^C NMR (126 MHz, CD_2_Cl_2_, δ): 168.31, 165.21 (dd, *J* = 260.3, 12.6 Hz), 162.11 (dd, *J* = 264.1, 10.1 Hz), 161.62, 161.57, 148.13, 146.64, 143.80, 141.61, 129.61, 124.84, 123.21, 122.37, 121.21 (d, *J* = 17.6 Hz), 120.08, 119.62 (d, *J* = 20.1 Hz), 103.40 (t, *J* = 26.4 Hz), 97.74, 97.33, 22.01; ^19^F NMR (470 MHz, CD_2_Cl_2_, δ): −104, −108; IR(KBr): ν = 2142 cm^−1^ (*v*(C≡C)); MALDI‐MS: *m/z* 794.1541 [M]^+^. Anal. calcd for C_39_H_25_AuF_4_N_2_·0.5MeOH: C 58.53, H 3.36, N 3.46; found: C 58.12, H 3.11, N 3.40.

7: 0.06 g of (C^^^N^PZ^^^^C)‐AuCl used. Yield: 0.06 g (68%). ^1^H NMR (400 MHz, CD_2_Cl_2_, δ): 8.83 (s, 2H), 8.22 (d, *J =* 2.0 Hz, 2H), 7.67 (d, *J =* 8.2 Hz, 2H), 7.36 (dd, *J =* 8.2, 2.0 Hz, 2H), 7.29–7.25 (m, 4H), 7.10 (d, *J* = 7.5 Hz, 4H), 7.03 (t, *J* = 7.3, 2H), 6.82 (s, 2H), 2.61 (s, 6H), 1.36 (s, 18H); ^13^C NMR (126 MHz, CD_2_Cl_2_, δ): 168.53, 157.92, 157.16, 148.32, 146.67, 144.88, 141.86, 138.85, 134.98, 129.76, 126.00, 125.01, 124.54, 123.35, 122.41, 120.76, 99.23, 98.30, 36.07, 31.55, 22.67; IR(KBr): ν = 2137 cm^−1^ (*v*(C≡C)); MALDI‐MS: *m/z* 835.3104 [M]^+^. Anal. calcd for C_46_H_44_AuN_3_: C 66.10, H 5.31, N 5.03; found: C 65.91, H 5.36, N 4.85.

8: 0.06 g of (C^^^N^PZ^^^^C)‐AuCl used. Yield: 0.05 g (62%). ^1^H NMR (500 MHz, CD_2_Cl_2_, δ): 8.83 (s, 2H), 8.19 (d, *J =* 2.0 Hz, 2H), 7.81 (d, *J =* 8.5 Hz, 2H), 7.67 (d, *J =* 8.5 Hz, 2H), 7.38 (dd, *J =* 8.5, 2.0 Hz, 2H), 7.34 (d, *J* = 8.5 Hz, 2H), 6.69–6.61 (m, 6H), 6.03–6.01 (m, 2H), 1.41 (s, 18H); ^13^C NMR (126 MHz, CD_2_Cl_2_, δ): 168.23, 157.89, 157.02, 144.69, 144.35, 138.77, 138.09, 134.85, 134.73, 134.21, 131.09, 127.19, 125.92, 124.68, 123.72, 121.71, 115.67, 113.77, 101.26, 93.56, 35.86, 31.30; IR(KBr): ν = 2154 cm^−1^ (*v*(C≡C)); MALDI‐MS: *m/z* 821.2573 [M]^+^. Anal. calcd for C_44_H_38_AuN_3_O·0.5CH_2_Cl_2_: C 61.84, H 4.55, N 4.86; found: C 61.55, H 4.47, N 4.82.

[CCDC 1879961 contains the supplementary crystallographic data for this paper. These data can be obtained free of charge from The Cambridge Crystallographic Data Centre via www.ccdc.cam.ac.uk/data_request/cif.]

## Conflict of Interest

The authors declare no conflict of interest.

## Supporting information

Supplementary
