# Supplementary material for: Thermally Stable Donor–Acceptor Type (Alkynyl)Gold(III) TADF Emitters Achieved EQEs and Luminance of up to 23.4% and 70 300 cd m−2 in Vacuum‐Deposited OLEDs
Source: Adv Sci (Weinh). 2019 Aug 6;6(18):1802297. doi: 10.1002/advs.201802297 (PMC6755518; doi:10.1002/advs.201802297)
Supplement: Supplementary file 1 — Supplementary [file ADVS-6-1802297-s001.pdf]

Copyright WILEY-VCH Verlag GmbH & Co. KGaA, 69469 Weinheim, Germany, 2019.

ADVANCED SCIENCE
Open Access

Supporting Information

for *Adv. Sci.*, DOI: 10.1002/advs. 201802297

Thermally Stable Donor–Acceptor Type (Alkynyl)Gold(III) TADF Emitters Achieved EQEs and Luminance of up to 23.4% and 70 300 cd m^{-2} in Vacuum-Deposited OLEDs

Dongling Zhou, Wai-Pong To, Yoonhyun Kwak, Yongsuk Cho, Gang Cheng, Glenna So Ming Tong,* and Chi-Ming Che**

Copyright WILEY-VCH Verlag GmbH & Co. KGaA, 69469 Weinheim, Germany, 2019.

Supporting Information

Thermally Stable Donor-Acceptor Type (Alkynyl)Gold(III) TADF Emitters Achieved EQEs and Luminance of up to 23.4 % and 70300 cd m^{-2} in Vacuum-Deposited OLEDs

Dongling Zhou, Wai-Pong To, Yoonhyun Kwak, Yongsuk Cho, Gang Cheng, Glenna So Ming Tong,* and Chi-Ming Che**

D. Zhou, Dr. W.-P. To, Dr. G. Cheng, Dr. G. S. M. Tong, Prof. Dr. C.-M. Che
State Key Laboratory of Synthetic Chemistry, Department of Chemistry, The University of Hong Kong, Pokfulam Road, Hong Kong SAR (China)
E-mail: ggcheng@hku.hk; tongsm@hku.hk; cmche@hku.hk

Dr. G. Cheng, Prof. Dr. C.-M. Che
HKU Shenzhen Institute of Research and Innovation, Shenzhen, Guangdong, 518053 (China)

Dr. G. Cheng
Sichuan Knowledge Express Institute for Innovative Technologies, World Financial Center, Gaoxin District, Chengdu 610041 (China)

Dr. Y. Kwak, Dr. Y. Cho
Organic Electronic Materials Lab., Samsung Advanced Institute of Technology, 130 Samsung-ro, Yeongtong-gu, Suwon-si, Gyeonggi-do, 16678, Korea

Table of Contents

Experimental section ······ S5

Synthesis and characterization ······ S5

Device performance evaluation of complex **6** by Samsung ······ S20

Comparison of device performance ······ S21

Computational details ······ S22

Table S1: Photophysical data of the gold(III) complexes ······ S6

Table S2: Photophysical data of **1-8** and [Ir(ppy)$_3$] in toluene at 60 and 20 ^{o}C ······ S12

Table S3: Emission lifetimes of **4** in 4 wt% PMMA thin films at different temperatures ······ S14

Table S4: Electrochemical data of **1-8** in DMF ······ S15

Table S5: Selected bond lengths and angles of **5** ······ S16

Table S6: Crystallographic data for complex **5** ······ S17

Table S7: Comparision of estimated LT$_{95}$ of gold(III)-OLEDs ······ S21

Table S8: HOMO-LUMO overlap at the optimized S$_1$ and T$_1$ excited states ······ S24

Table S9: Energy gaps and radiative decay rate constants at various δ of **2** ······ S25

Table S10: Radiative decay rate constants at optimized S$_1$ excited state geometrie ······ S26

Figure S1. Emission spectra of **3**, **6**, **7**, **8** in 4 wt% PMMA thin films, absorption spectra of **2** in different deoxygenated solvents, absorption spectra of **8** in oxygen-free hexane and toluene, at room temperature ······ S10

Figure S2. Emission spectra of **1-8** and Ir(ppy)$_3$ in toluene at different temperatures ······ S12

Figure S3. Emission spectra of **4** in 4 wt% PMMA thin films, percentage of long-lived component (> 100 μs) of emission decay of **4** in 4 wt% PMMA thin films, emission spectra of **3** in 4 wt% PMMA thin films, percentage of long-lived component of emission decay of **3** in 4 wt% PMMA thin films, at different temperatures ······ S13

Figure S4. Emission spectrum of **2** in 4 wt% PMMA thin film at room temperature and 77K ······ S14

Figure S5. TGA of complexes **1-8** ······ S15

Figure S6. Crystal structure of complex **5** ······ S16

Figure S7. Normalized PL spectra of **2**, **5** and **6** in TCTA:TPBi thin film with dopant concentration of 4 wt%; λ_{ex} = 320 nm ······ S18

Figure S8. Normalized EL spectra of OLEDs based on **2**, **5** and **6** with dopant concentration of 2, 4 and 8 wt% ······ S19

Figure S9. Spin density plots and the adiabatic energy difference of the optimized triplet excited states ······ S23

Figure S10. Vibrational frequencies of 2 at the optimized T_1^{cop} (top) and T_1^{perp} (bottom) geometries ···S23
Cartesian coordinates ···S27
References ···S37

Experimental Section

Synthesis and characterization

2,6-Bis(2,4-difluorophenyl)pyridine was prepared by Suzuki coupling reaction between 2,6-dibromopyridine and 2,4-difluorophenylboronic acid.[1] 2,6-Bis(4-*tert*-butyl phenyl)pyrazine was prepared by Suzuki coupling from 2,6-dichloropyrazine and 4-*tert*-butyl phenylboronic acid.[2b] The precursors (C^N^C)-AuCl / (C^N^{PZ}^C)-AuCl were synthesized by transmetallation from the corresponding mercury complexes.[2]

<u>Synthesis and characterization of *N*-substituted phenylacetylenes</u>

N-Substituted phenylacetylenes were prepared by using the corresponding phenyl bromides[3-6] according to the literature method for preparation of aryl acetylenes,[7] except minor changes in the synthesis of 4-ethynyl-3,5-dimethyl-*N*,*N*-diphenylaniline. Of the five *N*-substituted phenylacetylenes used in this work, 4-ethynyl-*N*,*N*-diphenylaniline is a known compound and commercially available.

3-Ethynyl-*N*,*N*-diphenylaniline: Yield: 94 %. ^{1}H NMR (500 MHz, CD_2Cl_2): δ 7.29-7.26 (m, 4H), 7.19 (t, *J* = 7.5 Hz, 1H), 7.15 (s, 1H), 7.11-7.03 (m, 8H), 3.06 (s, 1H); ^{13}C NMR (125 MHz, CD_2Cl_2): δ 148.49, 147.83, 129.77, 129.64, 126.99, 126.24, 124.99, 124.45, 123.71, 123.26, 83.71, 77.38. EI-MS (+ve, *m/z*): 269.1194 $[M]^+$.

10-(4-Ethynylphenyl)-9,9-dimethyl-9,10-dihydroacridine: Yield: 92 %. ^{1}H NMR (400 MHz, CD_2Cl_2): δ 7.78 (d, *J* = 8.3 Hz, 2H), 7.48 (dd, *J* = 7.4, 1.8 Hz, 2H), 7.34 (d, *J* = 8.7 Hz, 2H), 7.00-6.91 (m, 4H), 6.27 (dd, *J* = 7.7, 1.7 Hz, 2H), 3.26 (s, 1H), 1.68 (s, 6H); ^{13}C NMR (100 MHz, CD_2Cl_2): δ 142.20, 141.02, 135.04, 131.86, 130.58, 126.73, 125.66, 122.44, 121.12, 114.36, 83.18, 78.59, 36.31, 31.36. EI-MS (+ve, *m/z*): 309.1504 $[M]^+$.

10-(4-Ethynylphenyl)-10H-phenoxazine: Yield: 92 %. ^{1}H NMR (500 MHz, CD_2Cl_2): δ 7.73 (d, *J* = 8.5 Hz, 2H), 7.33 (d, *J* = 8.0 Hz, 2H), 6.69-6.64 (m, 4H), 6.61 (t, *J* = 8.0 Hz, 2H), 5.94 (d, *J* = 7.5 Hz, 2H), 3.25 (s, 1H); ^{13}C NMR (125 MHz, CD_2Cl_2): δ 144.30, 139.88, 135.22, 134.42, 131.38, 123.70, 122.76, 121.95, 115.78, 113.67, 83.00, 78.75. EI-MS (+ve, *m/z*): 283.0997 $[M]^+$.

4-Ethynyl-3,5-dimethyl-*N*,*N*-diphenylaniline: Prepared under the conditions similar to those reported in the literature[7] except that the reaction was heated at 50 ℃ for about 2 h. Yield: 90 %. ^{1}H NMR (500 MHz, CD_2Cl_2): δ 7.29-7.25 (m, 4H), 7.08-7.04 (m, 6H), 6.73 (s, 2H), 3.51 (s, 1H), 2.33 (s, 6H); ^{13}C NMR (125 MHz, CD_2Cl_2): δ 148.01, 147.83, 142.40, 129.70, 125.28, 123.67, 121.47, 115.82, 84.60, 81.75, 21.24. EI-MS (+ve, *m/z*): 297.1503 $[M]^+$.

Table S1: Photophysical data of the gold(III) complexes.[a]

	Absorption λ_{max} / nm (ε / 10^3 dm^3 mol^{-1} cm^{-1})	Medium	Emission λ_{max} / nm; τ / μs[b]	Φ_{em}[c]
**1**	319(6.52), 359(4.01), 378(3.97), 398(3.13)	Toluene	466, 495, 530; 0.34	0.002
		CH_2Cl_2 / MeOH / EtOH (1:1:4), 77 K	466, 498, 536; 218.22	
		Solid, rt	473, 504, 540; 0.20	
		Solid, 77 K	497, 534, 571; 141.27	
		4 wt% PMMA thin film, rt	468, 495, 530; 22.43	0.028
		4 wt% PMMA thin film, 77K	468, 495, 530 181.95	
**2**	294(31.78), 318(34.69), 379(7.39), 398(8.66), 426(br, 5.68)	Toluene	574; 0.78	0.60
	322(37.36), 382(8.17), 402(9.60), 430(br, 6.42)	1,2,4-Trichloro-benzene	592; 0.56	0.41
	320(34.20), 381(8.06), 400(9.55), 424(br, 5.70)	Chlorobenzene	626; 0.11	0.14

	321(33.72), 382(8.26), 401(9.71), 424(br, 5.70)	*o*-Dichloro-benzene	654; 0.062	0.06
		CH_2Cl_2 / MeOH / EtOH (1:1:4), 77 K	540; 4.77	
		Solid, rt	563; 0.61	
		Solid, 77 K	520, 552, 586; 97.01	
		4 wt% PMMA thin film, rt	577 0.85	0.88
**3**	295(34.90), 359(5.35), 379(5.43), 398(4.72)	Toluene	545; 1.25	0.21
		CH_2Cl_2 / MeOH / EtOH (1:1:4), 77 K	467, 500, 526; 119.26	
		Solid, rt	564; 0.95	
		Solid, 77 K	524; 17.59	
		4 wt% PMMA thin film, rt	546 3.78	0.29
**4**	320(15.24), 359(5.12), 379(6.17), 398(5.53)	Toluene	562; 0.80	0.49
		CH_2Cl_2 / MeOH / EtOH (1:1:4), 77 K	463, 494, 533; 188.92	
		Solid, rt	589; 0.25	
		Solid, 77 K	555; 3.30	

		4 wt% PMMA thin film, rt	560<br>1.43	0.67
**5**	290(20.45), 321 (17.27), 360(6.77), 379(5.90), 399(5.28)	Toluene	603;<br>0.84	0.57
		CH_2Cl_2 / MeOH / EtOH (1:1:4), 77 K	463, 494, 570;<br>176.24	
		Solid, rt	648;<br>0.30	
		Solid, 77 K	629;<br>1.99	
		4 wt% PMMA thin film, rt	602<br>0.98	0.51
**6**	319(26.74), 338(20.49), 380(4.99), 398(5.59), 435(br, 3.78)	Toluene	594;<br>0.33	0.25
		CH_2Cl_2 / MeOH / EtOH (1:1:4), 77 K	464, 500, 553;<br>140.58	
		Solid, rt	618;<br>0.25	
		Solid, 77 K	586;<br>37.65	
		1 wt% PMMA thin film, rt	572;<br>0.90	0.63
**7**	323 (34.09), 398 (4.63), 422 (7.26), 443 (8.15), 480 (br, 2.88)	Toluene	632;<br>0.20	0.024
		CH_2Cl_2 / MeOH / EtOH (1:1:4), 77 K	514, 553, 597;<br>88.57	
		Solid, rt	611;	

			0.084	
		Solid, 77 K	617; 5.46	
		4 wt% PMMA thin film, rt	605; 0.52	0.29
**8**	327 (23.16), 400 (4.31), 423 (6.09), 445 (5.75)	Toluene	625; 0.25	0.08
	326 (21.57), 352 (16.83), 402 (4.89), 423 (7.14), 448 (7.19)	Hexane	520, 558, 605 2.9	0.018
		CH_2Cl_2 / MeOH / EtOH (1:1:4), 77 K	519, 558, 599; 176.81	
		Solid, rt	596; 0.22	
		Solid, 77 K	607; 3.30	
		4 wt% PMMA thin film, rt	527, 570, 640; 0.33[d]	0.09

[a] For measurement in solution state, the absorption and emission data are obtained at 2 × 10^{-5} mol dm^{-3} and at room temperature. [b] Emission lifetime. [c] Emission quantum yield measured at 2 × 10^{-5} mol dm^{-3} at room temperature using 9,10-bis(phenylethynyl)-anthracene in degassed benzene (Φ = 0.85) as standard. [d] Emission lifetime was obtained at 640 nm.

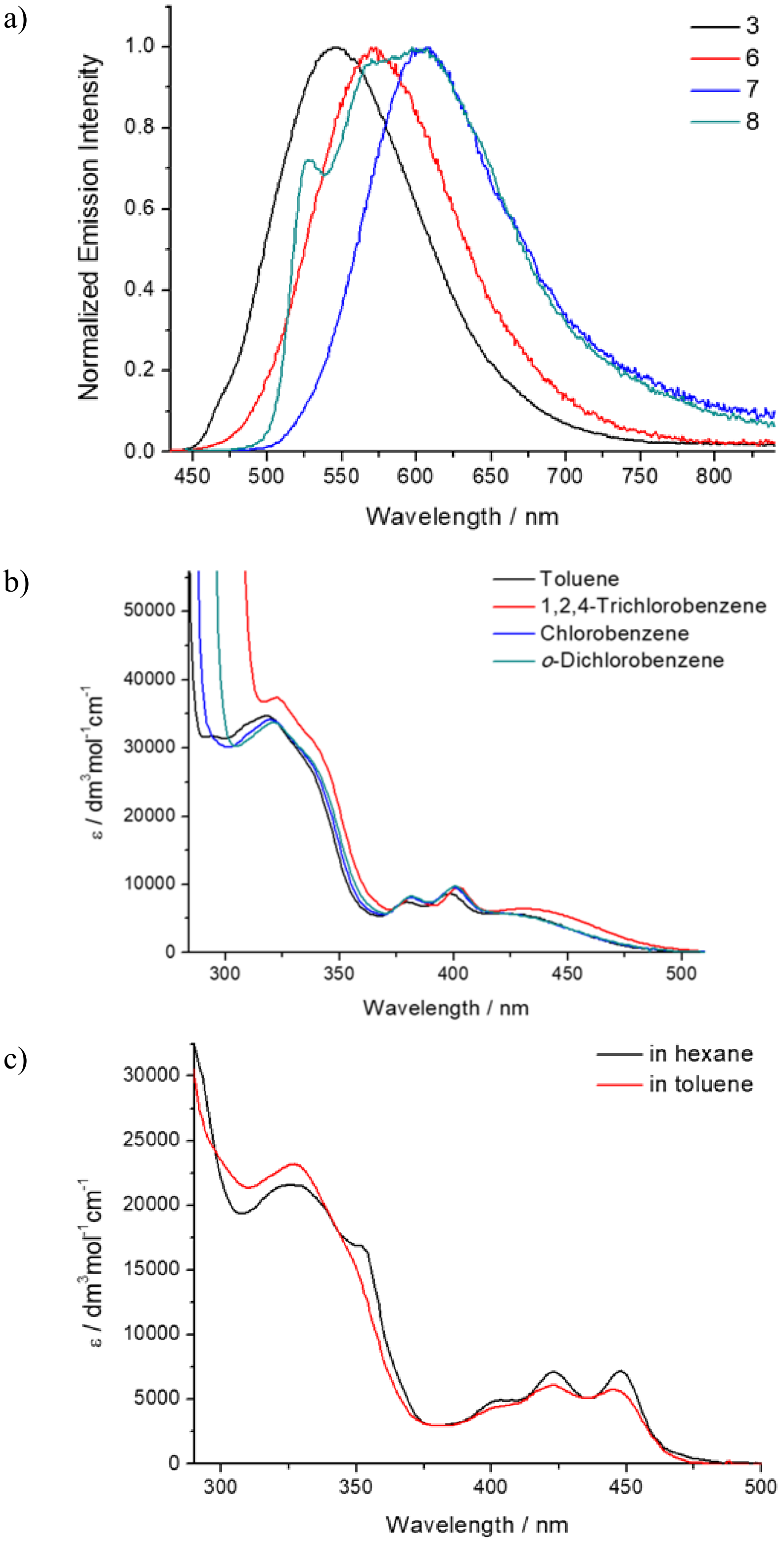

Figure S1. a) Emission spectra of **3**, **6**, **7**, **8** in 4 wt% PMMA thin films at room temperature; b) UV-vis absorption spectra of **2** in different deoxygenated solvents (2×10^{-5} mol dm^{-3}) at room temperature; c) UV-vis absorption spectra of **8** in oxygen-free hexane and toluene (2×10^{-5} mol dm^{-3}) at room temperature.

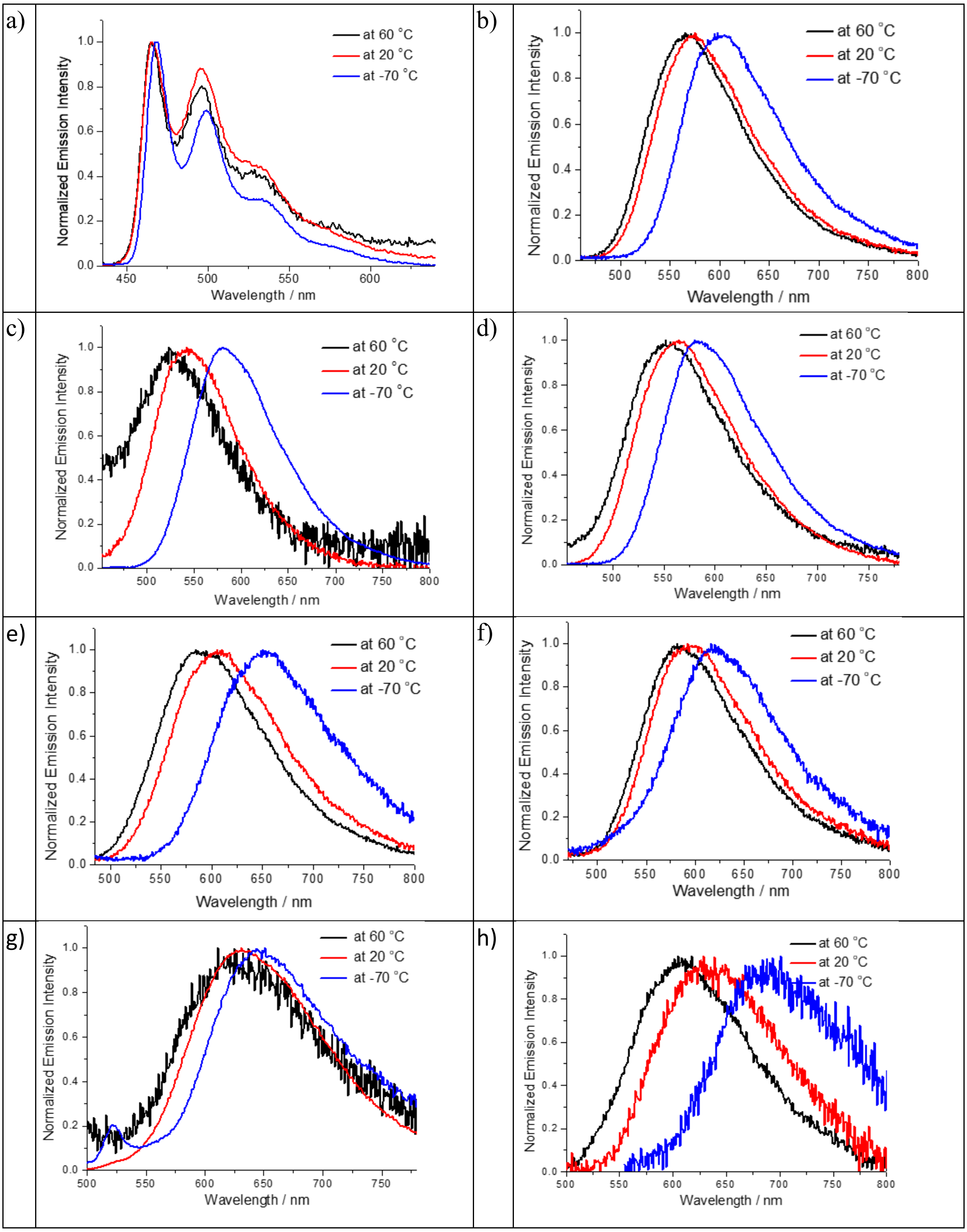

a)
Normalized Emission Intensity
Wavelength / nm
at 60 °C
at 20 °C
at -70 °C
b)
Normalized Emission Intensity
Wavelength / nm
at 60 °C
at 20 °C
at -70 °C
c)
Normalized Emission Intensity
Wavelength / nm
at 60 °C
at 20 °C
at -70 °C
d)
Normalized Emission Intensity
Wavelength / nm
at 60 °C
at 20 °C
at -70 °C
e)
Normalized Emission Intensity
Wavelength / nm
at 60 °C
at 20 °C
at -70 °C
f)
Normalized Emission Intensity
Wavelength / nm
at 60 °C
at 20 °C
at -70 °C
g)
Normalized Emission Intensity
Wavelength / nm
at 60 °C
at 20 °C
at -70 °C
h)
Normalized Emission Intensity
Wavelength / nm
at 60 °C
at 20 °C
at -70 °C

i)

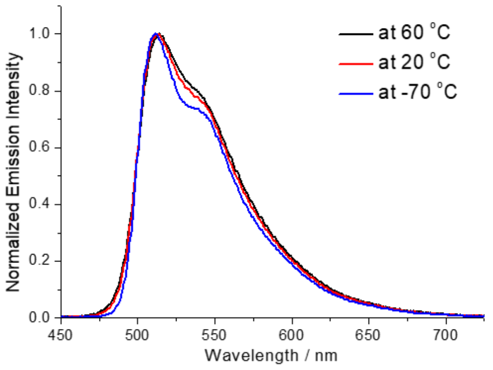

Figure S2. Emission spectra of a) complex **1**. b) complex **2**. c) complex **3**. d) complex **4**. e) complex **5**. f) complex **6**. g) complex **7**. h) complex **8**. i) [Ir(ppy)$_3$] in degassed toluene at 60 ^{o}C, 20 ^{o}C and -70 ^{o}C. (The emission spectra were measurement with Hamamatsu C11347 Quantaurus-QY Absolute PL quantum yields measurement system except for the spectra of **7** at 20 ^{o}C and -70 ^{o}C obtained by a HORIBA Fluorolog-3 spectrophotometer.)

Table S2: Photophysical data of **1**–**8** and [Ir(ppy)$_3$] in degassed toluene (2×10^{-5} mol/L) at 60 ^{o}C (first line) and 20 ^{o}C (second line, *italics*).[a]

complex	λ_{em} / nm	Φ / %	τ / µs	k_r / 10^5 s^{-1}	k_{nr} / 10^5 s^{-1}
**1**	466, 495, 530	0.1	< 0.1		
	466, 495, 530	*0.2*	*0.34*	*0.06*	*29.4*
**2**	567	61	0.57	10.7	6.84
	573	*59*	*0.78*	*7.56*	*5.26*
**3**	524	2	0.14	1.43	70.0
	545	*15*	*1.25*	*1.20*	*6.80*
**4**	552	14	0.19	7.37	45.3
	562	*47*	*0.80*	*5.88*	*6.63*
**5**	585	65	0.71	9.15	4.93
	605	*56*	*0.84*	*6.67*	*5.24*
**6**	580	33	0.29	11.4	23.1
	593	*28*	*0.33*	*8.48*	*21.8*
**7**	620	4	< 0.1		
	632	*2*	*0.2*	*1.00*	*49.0*
**8**	620	15	0.66	2.27	12.9
	625	*8*	*0.25*	*3.20*	*36.8*
**[Ir(ppy)$_3$]**	515, 545	86	1.33	6.47	1.05
	515, 545	*91*	*1.47*	*6.19*	*0.61*

[a] Emission quantum yields were measured with Hamamatsu C11347 Quantaurus-QY Absolute PL quantum yields measurement system.

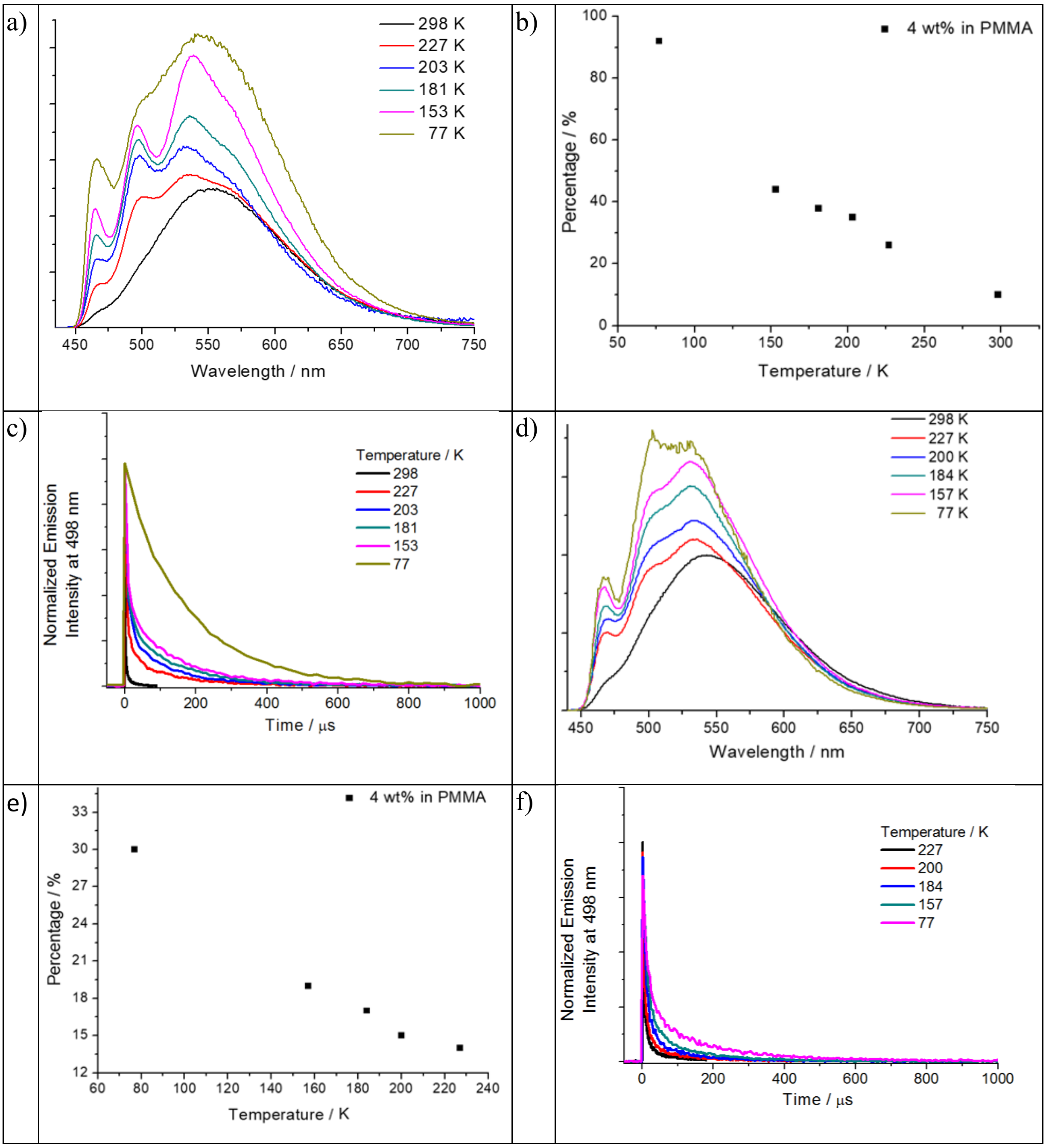

Figure S3. a) Emission spectra of **4** in 4 wt% PMMA thin films at different temperatures. b) Percentage of long-lived component (> 100 μs) of emission decay of **4** in 4 wt% PMMA thin films with decreasing temperature. c) Temperature-dependent emission intensity decay curves of the PMMA thin film doped with complex **4** (4 wt %). d) Emission spectra of **3** in 4 wt% PMMA thin films at different temperatures. e) Percentage of long-lived component of emission decay of **3** in 4 wt% PMMA thin films at different temperatures. f) Temperature-dependent emission intensity decay curves of the PMMA thin film doped with complex **3** (4 wt %).

Table S3: Emission lifetimes of **4** in 4 wt% PMMA thin films at different temperatures[a]

Complex	Temperature / K	$\tau_{465\ nm}$ / µs	$\tau_{498\ nm}$ / µs	
			long-lived component	short-lived component
**4**	298	23.09	11.37	1.38
	227	104.73	107.21	7.26
	203	121.00	129.64	11.22
	181	117.70	138.42	6.58
	153	146.73	160.63	12.82
	77	179.09	181.70	61.64

[a] Biexponential decays were fitted by using the formula of y=y0+A1e^(-x/t1)+A2e^(-x/t2) in Origin.

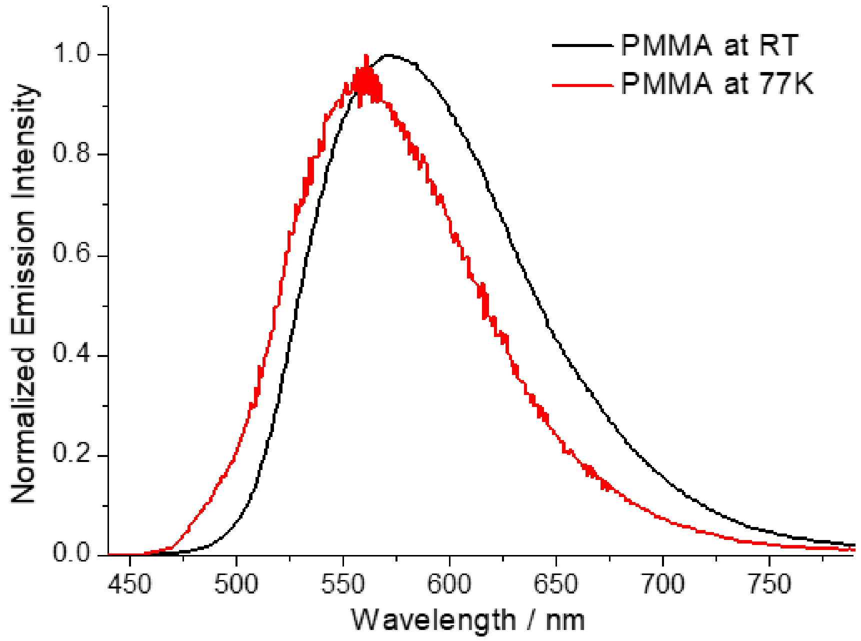

Figure S4. Emission spectrum of **2** (4 wt%) in PMMA thin films at room temperature and 77 K.

Table S4: Electrochemical data of **1**−**8** in DMF (0.1 mol dm^{-3} [$n$$Bu_4N$]$PF_6$)[a]

Complex	E (V vs. SCE)		
	oxidation (E_{pa})	1st reduction ($E_{1/2}$)	2nd reduction (E_{pc})
**1**	------	−1.21	−1.87
**2**	0.99	−1.21	−1.88
**3**	1.07	−1.21	−1.88
**4**	1.03	−1.20	−1.86
**5**	0.82[b]	−1.20	−1.86
**6**	0.94	−1.22	−1.90
**7**	0.94	−1.09	−1.84
**8**	0.78[b]	−1.06	−1.81

[a] In DMF with 0.1 mol dm^{-3} [$n$$Bu_4N$]$PF_6$ as the electrolyte at room temperature; glassy carbon as the working electrode; platinum wire as the counter electrode; scan rate 100 mV s^{-1}; E_{pa} refers to anodic peak potential for the irreversible oxidation waves; E_{pc} refers to cathodic peak potential for the irreversible reduction waves; $E_{1/2} = (E_{pa} + E_{pc})/2$. [b] Reversible oxidation couples.

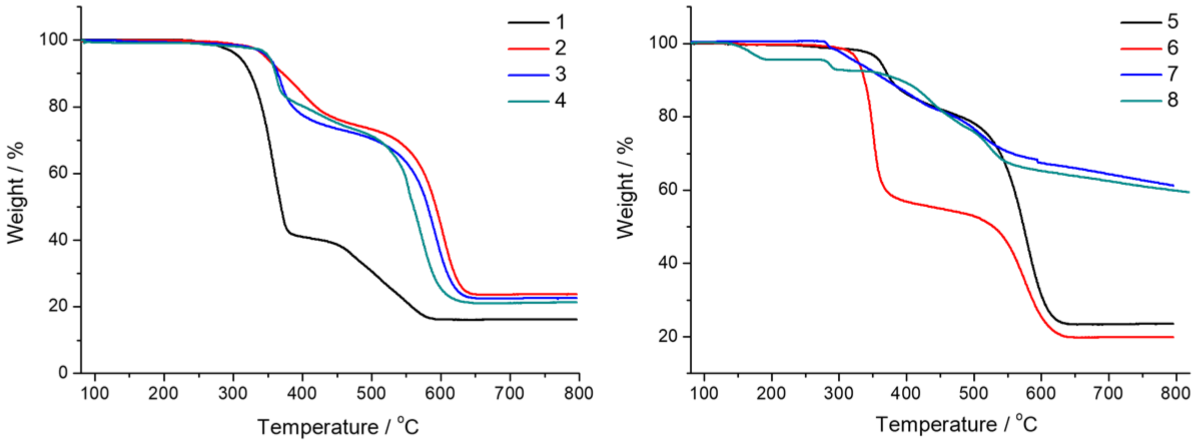

Figure S5. TGA of complexes **1**−**8**.

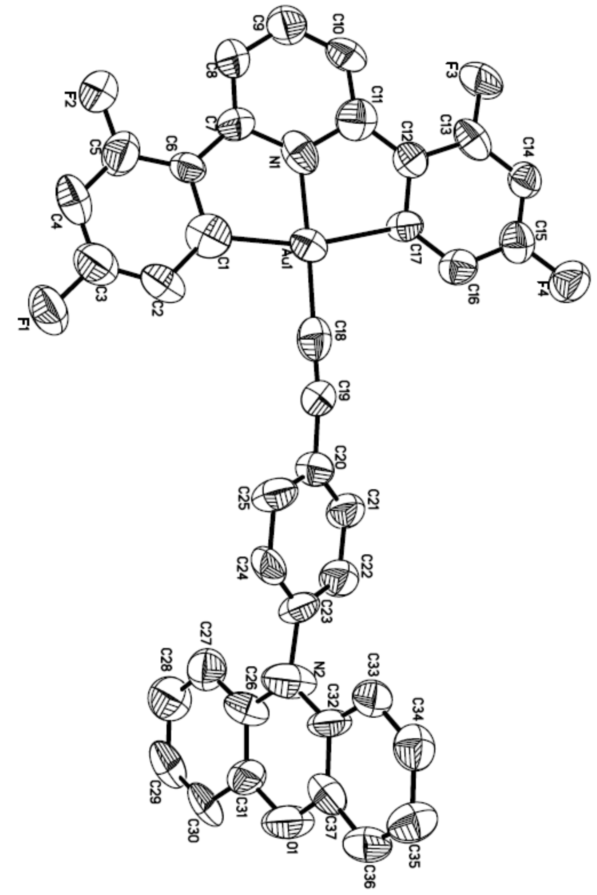

Figure S6. Crystal structure of complex **5**.

Table S5: Selected bond lengths and angles of **5**.

Selected bond lengths (Å)			
Au1–N1	1.958	Au1–C18	1.949
Au1–C1	2.060	C18–C19	1.220
Au1–C17	2.079	C19–C20	1.447
Selected bond angles (°)			
C1-Au1-C17	163.41	N1-Au1-C1	81.64
N1-Au1-C18	177.87	N1-Au1-C17	81.77

Table S6: Crystallographic data for complex **5**.[a]

Formula	$C_{37}H_{19}AuF_4N_2O \cdot C_6H_5F$
Formula Weight	876.61
Crystal system	Triclinic
Space group	P-1
a [Å]	10.117(2)
b [Å]	10.163(2)
c [Å]	18.243(4)
α [°]	77.568(9)
β [°]	74.635(8)
γ [°]	68.481(8)
V [Å^3]	1668.0(6)
ρ [g cm^{-3}]	1.745
Z	2
Radiation	Cu-Kα
2*θ*(max) [°]	106.094
T [K]	200
Total reflections	14997
Unique reflections	5782
Restraints	230
R_{int} [%]	11.83
R_1 [%] (all data)	10.10
wR_2 [%] (all data)	28.98
Goodness of fit	1.087
CCDC number	1879961

[a] The diffraction-quality crystal of **5** was obtained by slow evaporation of a solution of the complex in fluorobenzene.

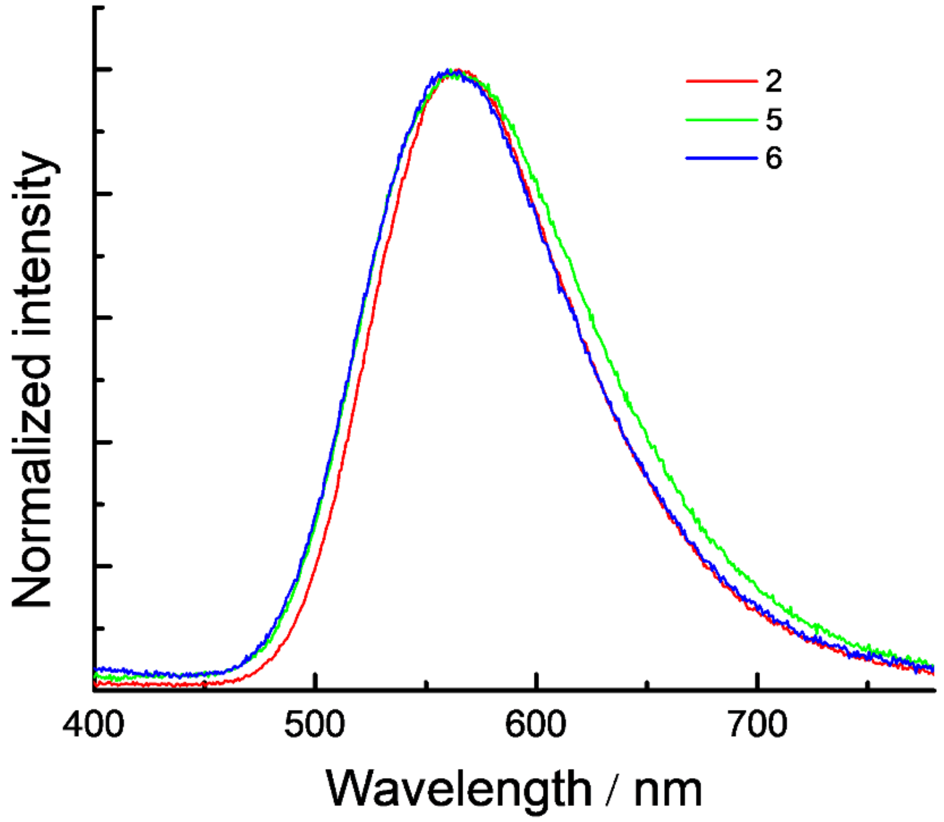

Figure S7. Normalized PL spectra of **2**, **5** and **6** in TCTA:TPBi thin film with dopant concentration of 4 wt%; λ_{ex} = 320 nm.

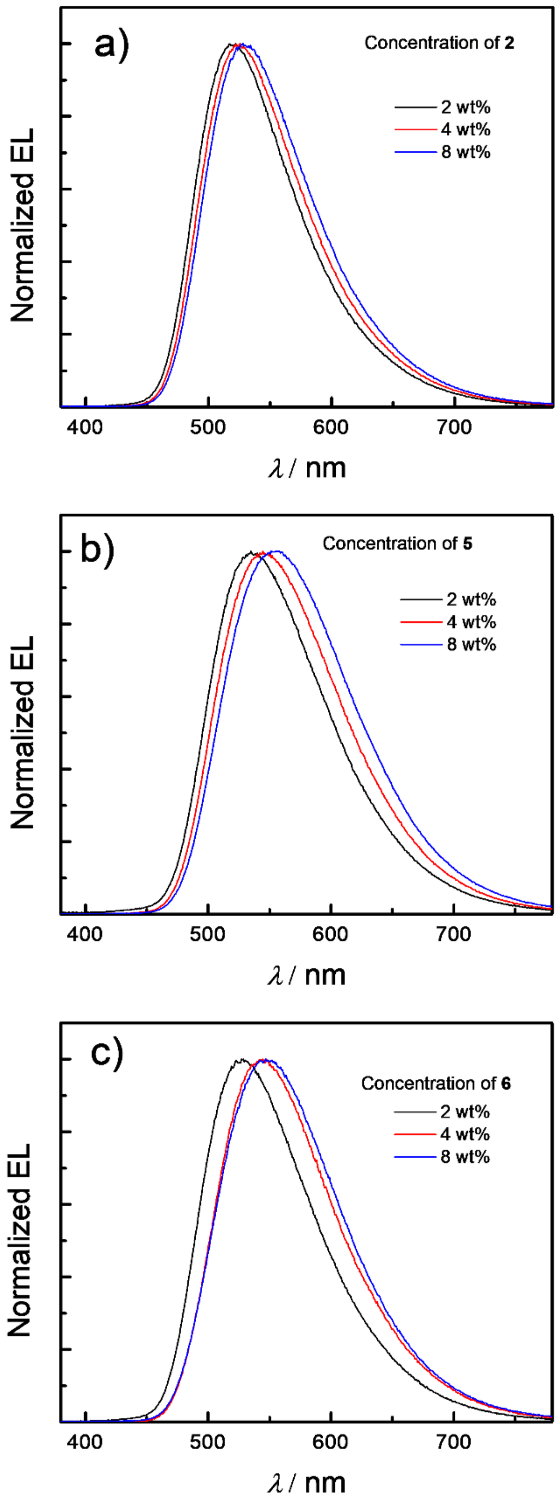

Figure S8. Normalized EL spectra of OLEDs based on a) **2**, b) **5** and c) **6** with dopant concentration of 2, 4 and 8 wt%.

Device performance evaluation of complex 6 by Samsung

The OLED performance of complex **6** was compared to a reference compound. “Au1” in the following figures represents complex **6** in this work. For device lifetime measurement, the initial current efficiency of the 5wt% gold-OLED is 53 cd/A and the corresponding EQE is 17 %. The LT_{95} of this device is approximately 0.3 hour. By using the formula $LT(L_1) = LT(L_0)\times(L_0/L_1)^{1.7}$ where L_0 refers to initial luminance and L_1 refers to desired luminance, LT_{95} at luminance of 1000 cd m^{-2} and 100 cd m^{-2} were respectively estimated to be about 10 and 500 hours.[8]

Sample Description	Op. V	Op. J	MAX cd/A	cd/A @Op.	Max EQE	EQE @Op.	Roll-Off	λmax (nm)	FWHM	CIE_x @Op.	V @ 5mA/cm2
Host1/Reference 12wt%	4.29	10.15	100%	100%	100%	100%	8%	527	67.70	0.359	3.74
**Host1/Au1 5wt%**	**4.83**	**15.01**	**66.4%**	**67.8%**	**74.9%**	**76.9%**	**6%**	**546**	**100.60**	**0.381**	**3.89**
**Host1/Au1 10wt%**	**4.89**	**14.72**	**68.9%**	**69.0%**	**78.8%**	**78.2%**	**8%**	**556**	**99.36**	**0.414**	**4.01**

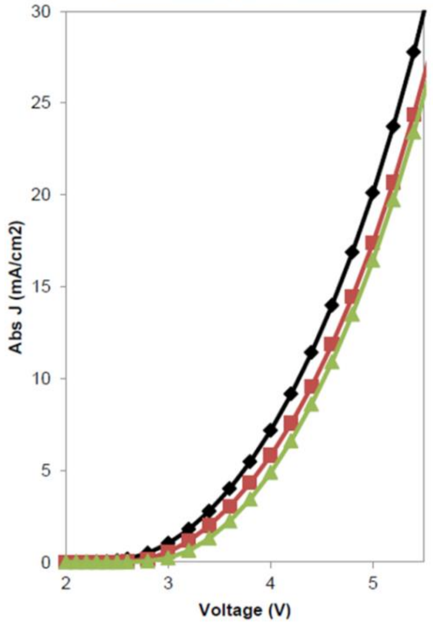

Current density-voltage curves of devices.

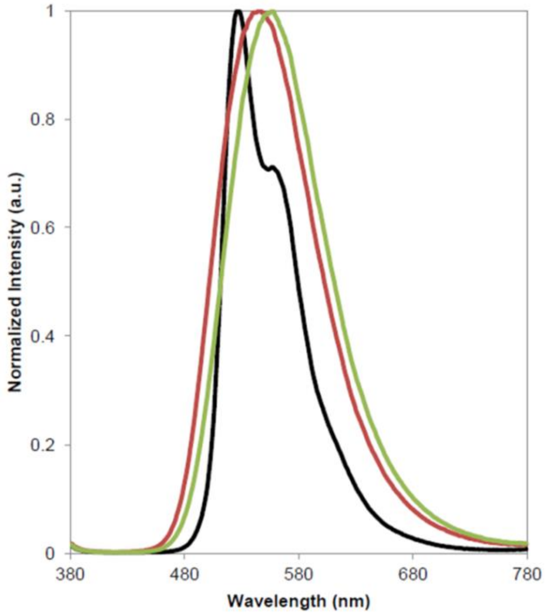

EL spectra of devices.

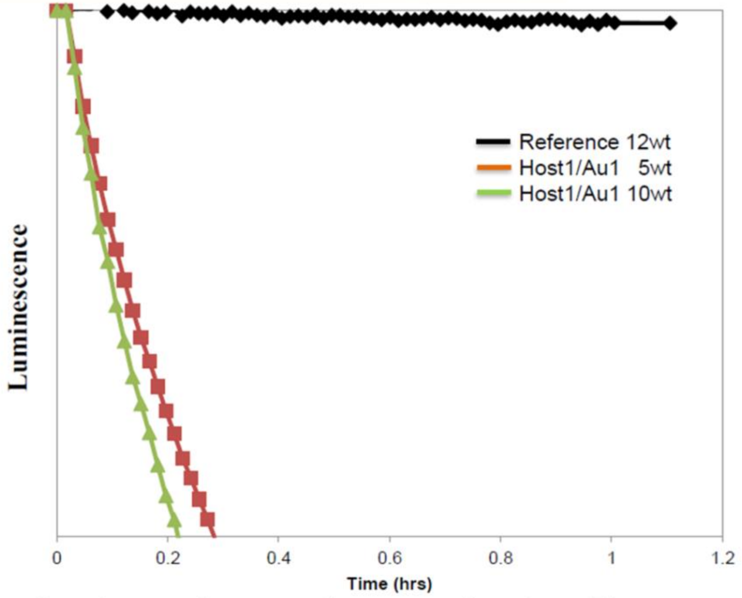

Luminance decay against operation time. The upper and lower boundary of the y-axis respectively represent the initial luminance and 95% of initial luminance.

Comparison of device performance

The performance of the OLED prepared with **6** and evaluated by Samsung was compared to gold(III)-OLEDs reported recently.[9]

Table S7: Comparison of estimated LT_{95} of gold(III)-OLEDs

Gold(III) emitter	EQE	Initial luminance / cd m^{-2}	Lifetime / hours		
			LT_{95}	LT_{95} at 1000 cd m^{-2} [e]	LT_{95} at 100 cd m^{-2} [e]
Complex **6** in this work	17 %	7955	0.3	10	511
Complex **3** in reference 9	15 %[a]	4435[c]	0.8[d]	10	504
Complex **4** in reference 9	5 %[b]	1775[c]	4[d]	11	532
Complex **5** in reference 9	4 %[b]	1176[c]	60[d]	79	3961

[a] Value estimated from Figure S11 with current density of 10 mA cm^{-2} in reference 9.

[b] Value estimated from Figure 5d with current density of 20 mA cm^{-2} in reference 9.

[c] Data taken from Table S11 in reference 9.

[d] Value estimated from Figure 6 in reference 9.

[e] LT_{95} at luminance of 1000 cd m^{-2} and 100 cd m^{-2} were respectively estimated by using the formula $LT(L_1) = LT(L_0)\times(L_0/L_1)^{1.7}$ where L_0 refers to initial luminance and L_1 refers to desired luminance.

Computational details

In this work, the hybrid density functional, M06,[10] was employed for all calculations using the program package G09.[11] The 6-31G* basis set[12] is used for all atoms except Au, which is described by the Stuttgart relativistic pseudopotential and its accompanying basis set (ECP60MWB).[13] Solvent effect was also included by means of the polarizable continuum model (PCM)[14] and default parameters are used for the solvent, toluene (refractive index η = 1.4969). No symmetry constraints were applied in geometry optimizations. For the singlet ground state (S_0), the restricted density functional theory (RDFT) formalism was employed. For the triplet excited states, both unrestricted DFT (UDFT) and TDDFT were employed. The singlet excited state was optimized using TDDFT. Frequency calculations were performed on the optimized structures to ensure that they are minimum energy structures by the absence of imaginary frequency (i.e. NImag = 0). Stability calculations were also performed for all the optimized structures to ensure that all the wavefunctions obtained are stable.

The excited state energies at the optimized excited state geometries were computed using a state-specific approach.[15] The radiative decay rate constants, k_r, were computed at the optimized S_1 and T_1 excited state geometries. Detailed procedures for radiative decay rate constant calculations were reported in previous works.[16] Relative excited state energy gaps were computed using TDDFT within the Tamm-Dancoff approximation (TDA)[17] to avoid the triplet instability problems.[18]

<u>TADF rate constant calculations</u>

The rate constant of TADF ($k_{r,avg}$) is estimated assuming Boltzmann equilibrium of the S_1 and T_1 excited states:[19]

$$k_{r,avg} = \frac{3k_r(T_1)+k_r(S_1)\exp(-\Delta E(S_1-T_1)/k_BT)}{3+\exp(-\Delta E(S_1-T_1)/k_BT)} \quad \text{(S1)}$$

$k_r(T_1)$ = radiative decay rate of the $T_1 \rightarrow S_0$ transition

$k_r(S_1)$ = radiative decay rate of the $S_1 \rightarrow S_0$ transition

$\Delta E(S_1–T_1)$ = energy gap between the S_1 and T_1 excited states

^{3}LLCT excited state was optimized by both TDDFT and UDFT methods, but the geometries obtained from the two methods differ in two aspects:

(1) δ = 178° from TDDFT optimization but 5.41° from UDFT optimization

(2) TDDFT optimization from both coplanar and perpendicular starting geometries gives a nearly coplanar conformation; similar UDFT optimizations give two local T_1 minima: δ = 5.41° and 101°.

We have also computed the ^{3}IL(C^N^C) energy using the UDFT method to gauge the relative energies of the ^{3}IL(C^N^C) and ^{3}LLCT minima. Spin-density plot at the optimized ^{3}IL(C^N^C) geometry confirmed that the computed energy corresponds to the ^{3}IL(C^N^C) excited state. The relative adiabatic energy between the ^{3}IL(C^N^C) and the T_1^{cop} optimized geometry is ~2400 cm^{-1}.

	^{3}IL(C^N^C)	T_1^{cop} (^{3}LLCT)	T_1^{perp} (^{3}LLCT)
	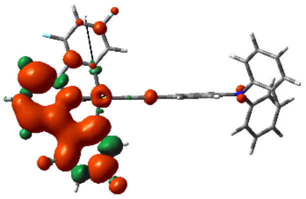	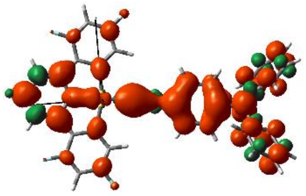	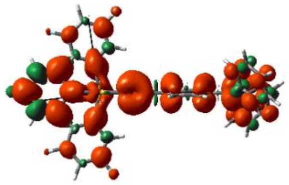
ΔE_{ad} / cm^{-1}	2439	0	254

Figure S9. Spin density plots and the adiabatic energy difference (ΔE_{ad}) of the optimized triplet excited states. ΔE_{ad} is the adiabatic energy relative to the optimized T_1^{cop} excited state.

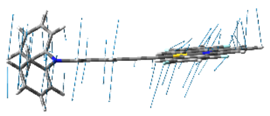	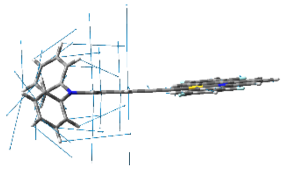	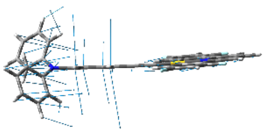	
ν_3 = 14.57 cm^{-1}	ν_8 = 48.46 cm^{-1}	ν_{13} = 67.58 cm^{-1}	

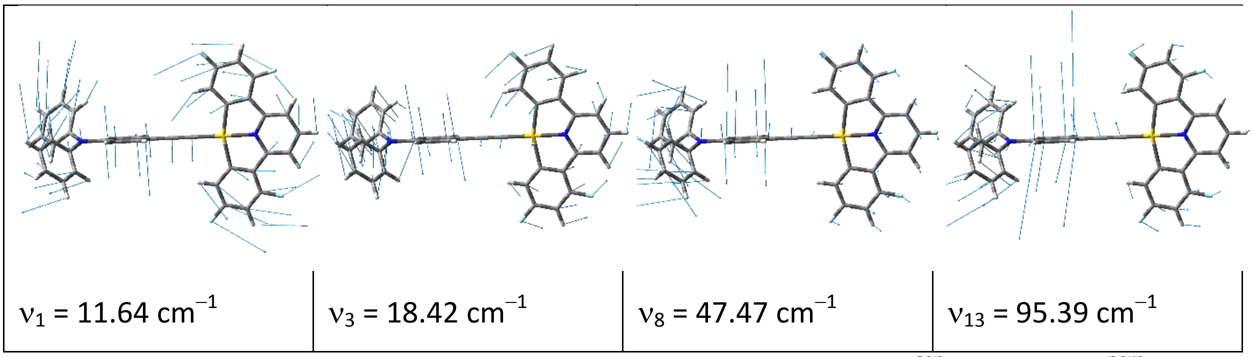

Figure S10. Vibrational frequencies of complex **2** at the optimized T_1^{cop} (top) and T_1^{perp} (bottom) geometries.

The HOMO-LUMO overlaps were computed using the program Multiwfn.[20] Details about these calculations could be found in the Multiwfn manual (version 3.4).

Table S8: HOMO-LUMO overlap at the optimized S_1 and T_1 excited states**.**

	S_1	T_1^{cop}	T_1^{perp}
Overlap	0.079	0.28	0.12

We have further computed the ΔE_{ST}, $k_r^P(T_1)$, $k_r^F(S_1)$, and $k_{r,avg}$ at various dihedral angle δ. Here, we fixed the molecular conformation at the optimized T_1^{cop} geometry and only δ was changed from ~5° at the optimized T_1^{cop} geometry to ~105°. Table S9 lists the computed decay rate constants and energy gap at different δ values. At all the dihedral angles considered, $k_r^P(T_1)$ is never greater than 10^4 s^{-1}; thus, phosphorescence alone could hardly explain the observed fast radiative decay rate constant of the complex. After invoking the TADF mechansim, the thermally averaged $k_{r,avg}$ would be of the order 10^5 s^{-1}. Hence, the emission mechanism of complex **2** is most consistent with a TADF mechanism.

Table S9. Energy gaps and radiative decay rate constants at various δ of complex **2**.

δ / °	λ_{em} [a] / nm	ΔE_T [c] / cm^{-1}	ΔE_{ST} / cm^{-1}	$k_{r\text{-phosphorescence}}$ / s^{-1}	$k_{r\text{-fluorescence}}$ / s^{-1}	$k_{r,avg}$ / s^{-1}
5.4	561		2660	2.70×10^2	4.34×10^8	5.13×10^2
35	560	186<br>–450	2246	9.95×10^2	3.06×10^8	2.35×10^3
55	560	402<br>–1168	1535	1.33×10^3	1.59×10^8	2.59×10^4
85	557	683<br>–2236	83	1.87×10^3	6.19×10^6	1.12×10^6
95	553	699<br>–2275	8	1.80×10^3	1.50×10^6	3.65×10^5
105	553	660<br>–2058	342	1.65×10^3	2.39×10^7	1.36×10^6
101 [b]	570	254<br>–2363	182	1.43×10^3	1.04×10^7	1.23×10^6

[a] λ_{em} corresponds to the vertical transition energy ($E(T_1, \delta) - E(S_0, \delta)$)

[b] the last line is the computed energy gaps and rate constants at the optimized T_1^{perp} geometry.

[c] $\Delta E_T = E(T_1, \delta) - E(T_1^{cop})$; first line is derived from UDFT and the second line from TDDFT

[d] $\Delta E_{ST} = E(S_1, \delta) - E(T_1, \delta)$

As TDDFT and UDFT are two different methods and the relative energies of the S_1 and T_1 minima could not be compared since they are determined by two different methods. Hence, the energy of the S_1 excited state is estimated in the following way:

First, the energy gap between the ^{3}LLCT and ^{1}LLCT excited states, both determined from SS-PCM TDDFT, were determined (ΔE_{ST}^{SS}) at the optimized T_1^{perp} geometry ($Q_{eq}^{T1\text{-}perp}$) since the optimized S_1 geoemtry also has the dihedral angle close to 90°. Then, the energy difference between the SS-PCM corrected energies of the ^{1}LLCT excited state were computed at the optimized ^{1}LLCT geoemtry (Q_{eq}^{S1}) and the optimzied T_1^{perp} geometry (ΔE_S^{SS}); the energy of the optimized S_1 excited state, $E_{UDFT}(^1LLCT)$, is then estimated by

$$E_{UDFT}(^1LLCT) = E_{UDFT}(^3LLCT) + \Delta E_{ST}^{SS} + \Delta E_S^{SS}$$

$$\Delta E_{ST}^{SS} = E^{SS}(S_1; Q_{eq}^{T1\text{-}perp}) - E^{SS}(T_1^{perp}; Q_{eq}^{T1\text{-}perp})$$

$$\Delta E_S^{SS} = E^{SS}(S_1; Q_{eq}^{S1}) - E^{SS}(S_1; Q_{eq}^{T1\text{-}perp})$$

where $E_{UDFT}(^3LLCT)$ is the energy of the ^{3}LLCT excited state optimized using the UDFT method at $Q_{eq}^{T1\text{-}perp}$.

Table S10. Radiative decay rate constants at optimized S_1 excited state geometries.

	λ_{em} / nm	$k_{r\text{-phosphorescence}}$ / s^{-1}	$k_{r\text{-fluorescence}}$ / s^{-1}	$k_{r\text{-TADF}}$ / s^{-1}
S_1	579[a)]	4.16×10^2	6.80×10^2	4.87×10^2

[a)] Vertical emission energy computed using the estimated ^{1}LLCT emission energy.

Cartesian coordinates

Table S11: Optimized S_0^{cop} geometry of complex **2**.

Center Number	Atomic Number	Atomic Type	Coordinates (Angstroms) X	Y	Z
1	79	0	-0.00456	0.047611	0.012447
2	6	0	0.019203	-0.25417	2.076171
3	7	0	1.965492	0.052946	0.3191
4	6	0	0.647762	0.26958	-1.955552
5	6	0	-1.06219	-0.36974	2.949201
6	1	0	-2.0914	-0.33561	2.595578
7	6	0	-0.8244	-0.5391	4.302206
8	6	0	0.460723	-0.5878	4.830516
9	1	0	0.633412	-0.71343	5.895327
10	6	0	1.518792	-0.46605	3.95335
11	6	0	1.357878	-0.30125	2.57073
12	6	0	2.43211	-0.18484	1.604883
13	6	0	3.798515	-0.28926	1.801161
14	1	0	4.185075	-0.46412	2.799163
15	6	0	4.669931	-0.17605	0.711287
16	6	0	4.170332	0.030756	-0.580273
17	1	0	4.844532	0.099226	-1.426443
18	6	0	2.805322	0.136966	-0.783455
19	6	0	2.073851	0.309606	-2.022727
20	6	0	-0.11993	0.410786	-3.111113
21	1	0	-1.20798	0.383168	-3.083863
22	6	0	0.515294	0.587313	-4.327904
23	6	0	1.899985	0.642032	-4.44191
24	1	0	2.385303	0.789663	-5.402137
25	6	0	2.644461	0.504613	-3.288215
26	1	0	5.742883	-0.26526	0.866568
27	6	0	-1.94241	0.10822	-0.272962
28	6	0	-3.16938	0.102474	-0.455808
29	6	0	-4.54529	0.088421	-0.655601
30	6	0	-5.2767	1.296912	-0.819485
31	1	0	-4.73247	2.238549	-0.809628
32	6	0	-6.63235	1.283607	-1.008464
33	1	0	-7.17059	2.215818	-1.159396
34	6	0	-7.33966	0.053269	-1.056406
35	6	0	-6.61756	-1.15906	-0.89698
36	1	0	-7.15281	-2.10513	-0.899054
37	6	0	-5.26365	-1.13825	-0.698433
38	1	0	-4.71633	-2.0666	-0.551319
39	6	0	-9.5125	1.161353	-0.931706
40	6	0	-9.40978	1.761265	0.325505
41	1	0	-8.7025	1.368927	1.054664
42	6	0	-10.2255	2.842179	0.635396
43	1	0	-10.1486	3.307304	1.615862

44	6	0	-11.1451	3.316958	-0.296315
45	1	0	-11.7837	4.161934	-0.047654
46	6	0	-11.2496	2.707699	-1.545061
47	1	0	-11.9644	3.079177	-2.276284
48	6	0	-10.4372	1.629259	-1.867342
49	1	0	-10.5061	1.14849	-2.841733
50	6	0	-9.35233	-1.1082	-1.803097
51	6	0	-10.5008	-1.60396	-1.183085
52	1	0	-10.8675	-1.12931	-0.274526
53	6	0	-11.1515	-2.70212	-1.728824
54	1	0	-12.0418	-3.09503	-1.242468
55	6	0	-10.6644	-3.30257	-2.888143
56	1	0	-11.1784	-4.16212	-3.31305
57	6	0	-9.5228	-2.79887	-3.50654
58	1	0	-9.14685	-3.25599	-4.419364
59	6	0	-8.8656	-1.69831	-2.971642
60	1	0	-7.98408	-1.28365	-3.458237
61	7	0	-8.69875	0.035406	-1.255661
62	9	0	-1.86267	-0.66394	5.143829
63	9	0	2.754707	-0.50833	4.480482
64	9	0	-0.21961	0.71131	-5.444411
65	9	0	3.981322	0.568803	-3.414098

Table S12: Optimized S_0^{perp} geometry of complex **2**.

Center Number	Atomic Number	Atomic Type	Coordinates (Angstroms) X	Y	Z
1	79	0	-0.0014	0.00383	-0.02826
2	6	0	-0.03039	0.009463	2.054449
3	7	0	1.999727	-0.00123	0.317684
4	6	0	0.671898	-0.00334	-2.0004
5	6	0	-1.11583	0.015675	2.91576
6	1	0	-2.13778	0.018065	2.540299
7	6	0	-0.88861	0.019128	4.287769
8	6	0	0.384854	0.016607	4.832273
9	1	0	0.543908	0.019316	5.906243
10	6	0	1.456388	0.010546	3.95719
11	6	0	1.297687	0.006866	2.569621
12	6	0	2.407055	0.000962	1.608617
13	6	0	3.779048	-0.00237	1.860292
14	1	0	4.152663	-0.00065	2.876173
15	6	0	4.651925	-0.00784	0.778498
16	6	0	4.19469	-0.01003	-0.53415
17	1	0	4.889025	-0.01427	-1.36463
18	6	0	2.818226	-0.00651	-0.76043
19	6	0	2.096014	-0.00775	-2.03861
20	6	0	-0.0601	-0.00463	-3.1769
21	1	0	-1.14863	-0.00157	-3.16743
22	6	0	0.615246	-0.01008	-4.39283
23	6	0	1.997668	-0.01442	-4.47669

24	1	0	2.509113	-0.01873	-5.43442
25	6	0	2.712529	-0.01319	-3.2923
26	1	0	5.724146	-0.01047	0.964522
27	6	0	-1.94442	0.008764	-0.35181
28	6	0	-3.15309	0.012214	-0.53193
29	6	0	-4.56552	0.015963	-0.73145
30	6	0	-5.26833	1.219802	-0.89501
31	1	0	-4.71808	2.159353	-0.88495
32	6	0	-6.64031	1.224471	-1.08368
33	1	0	-7.16824	2.166615	-1.22148
34	6	0	-7.36146	0.02421	-1.09885
35	6	0	-6.66725	-1.18059	-0.93216
36	1	0	-7.21925	-2.11911	-0.93699
37	6	0	-5.29307	-1.18378	-0.75978
38	1	0	-4.76542	-2.12697	-0.62687
39	6	0	-9.55389	1.029205	-0.66574
40	6	0	-9.29253	1.438266	0.646223
41	1	0	-8.47319	0.975854	1.194488
42	6	0	-10.0707	2.424991	1.238651
43	1	0	-9.85416	2.732401	2.260648
44	6	0	-11.1287	3.004782	0.543432
45	1	0	-11.7399	3.772991	1.013235
46	6	0	-11.3962	2.591343	-0.75916
47	1	0	-12.2161	3.041022	-1.31718
48	6	0	-10.6121	1.618033	-1.36572
49	1	0	-10.8138	1.304288	-2.38885
50	6	0	-9.38139	-0.97871	-2.0561
51	6	0	-10.571	-1.57332	-1.62276
52	1	0	-11.009	-1.25702	-0.67724
53	6	0	-11.1834	-2.55353	-2.39367
54	1	0	-12.1093	-3.00696	-2.04301
55	6	0	-10.6133	-2.96727	-3.59472
56	1	0	-11.0919	-3.74079	-4.19225
57	6	0	-9.4246	-2.38161	-4.02327
58	1	0	-8.97158	-2.69057	-4.96407
59	6	0	-8.81499	-1.38768	-3.2677
60	1	0	-7.89351	-0.9198	-3.61077
61	7	0	-8.76107	0.028691	-1.27797
62	9	0	-1.92954	0.02511	5.119112
63	9	0	2.682789	0.008335	4.496956
64	9	0	-0.08561	-0.01132	-5.52567
65	9	0	4.049062	-0.01755	-3.38726

Table S13: Optimized S_1^{perp} geometry of complex **2**.

Center Number	Atomic Number	Atomic Type	Coordinates (Angstroms) X	Y	Z
1	79	0	0.001343	0.028194	-0.00713
2	6	0	0.089976	-1.27206	1.6122
3	7	0	1.960409	0.058547	0.315524

4	6	0	0.564591	1.340174	-1.52018
5	6	0	-0.95356	-1.94955	2.240175
6	1	0	-1.98445	-1.84154	1.90707
7	6	0	-0.6767	-2.78175	3.310581
8	6	0	0.61494	-2.96873	3.788801
9	1	0	0.821384	-3.62413	4.629661
10	6	0	1.638112	-2.29159	3.155493
11	6	0	1.435666	-1.43356	2.066679
12	6	0	2.470518	-0.70007	1.361213
13	6	0	3.833908	-0.66407	1.590278
14	1	0	4.257065	-1.24431	2.402249
15	6	0	4.659216	0.121195	0.775753
16	6	0	4.115219	0.877404	-0.2703
17	1	0	4.754392	1.485964	-0.89986
18	6	0	2.752593	0.851875	-0.50312
19	6	0	1.97824	1.552918	-1.51089
20	6	0	-0.23733	1.97966	-2.4641
21	1	0	-1.31578	1.833106	-2.49125
22	6	0	0.345794	2.824501	-3.39236
23	6	0	1.714619	3.063989	-3.41948
24	1	0	2.161164	3.731935	-4.15002
25	6	0	2.496141	2.423621	-2.4784
26	1	0	5.730529	0.145334	0.958078
27	6	0	-1.94479	-0.00122	-0.30995
28	6	0	-3.16721	-0.01648	-0.48393
29	6	0	-4.55064	-0.03144	-0.67037
30	6	0	-5.38338	0.915004	-0.01617
31	1	0	-4.91743	1.670209	0.61178
32	6	0	-6.74267	0.896353	-0.18365
33	1	0	-7.36459	1.644134	0.301491
34	6	0	-7.34559	-0.06795	-1.03189
35	6	0	-6.52113	-1.00972	-1.70004
36	1	0	-6.97904	-1.77009	-2.3272
37	6	0	-5.16403	-0.99383	-1.51549
38	1	0	-4.53443	-1.73451	-2.002
39	6	0	-9.59354	0.409462	-0.20788
40	6	0	-9.41206	0.049681	1.130458
41	1	0	-8.59683	-0.61743	1.404971
42	6	0	-10.2885	0.533583	2.092416
43	1	0	-10.1506	0.250526	3.133493
44	6	0	-11.3466	1.360941	1.724786
45	1	0	-12.0332	1.734866	2.481017
46	6	0	-11.5301	1.705575	0.38695
47	1	0	-12.3547	2.353135	0.097553
48	6	0	-10.6588	1.231538	-0.58327
49	1	0	-10.7867	1.502046	-1.62979
50	6	0	-9.28725	-0.62541	-2.39525
51	6	0	-10.3316	-1.54721	-2.30094
52	1	0	-10.6793	-1.86825	-1.32061
53	6	0	-10.9	-2.05362	-3.46142

54	1	0	-11.7057	-2.78089	-3.39076
55	6	0	-10.4364	-1.64026	-4.70896
56	1	0	-10.8858	-2.03941	-5.61557
57	6	0	-9.40097	-0.71322	-4.79639
58	1	0	-9.04593	-0.37732	-5.76807
59	6	0	-8.82445	-0.19921	-3.64232
60	1	0	-8.02903	0.541874	-3.69944
61	7	0	-8.71029	-0.09193	-1.20567
62	9	0	-1.68045	-3.43775	3.915936
63	9	0	2.880738	-2.48682	3.630806
64	9	0	-0.42662	3.441774	-4.30162
65	9	0	3.817617	2.67003	-2.51829

Table S14: Optimized T_2 (LLCT) geometry of complex **2**.

Center Number	Atomic Number	Atomic Type	Coordinates (Angstroms) X	Y	Z
1	79	0	-0.01257	-0.03931	-0.02706
2	6	0	0.039979	-1.24969	1.659507
3	7	0	1.959572	-0.03059	0.296673
4	6	0	0.564439	1.175989	-1.6095
5	6	0	-1.01109	-1.87083	2.323676
6	1	0	-2.03994	-1.75828	1.984337
7	6	0	-0.7417	-2.64983	3.438097
8	6	0	0.546196	-2.83557	3.92232
9	1	0	0.742343	-3.44832	4.796945
10	6	0	1.580461	-2.21201	3.250694
11	6	0	1.381512	-1.41335	2.118945
12	6	0	2.436358	-0.73517	1.371824
13	6	0	3.805366	-0.71571	1.60943
14	1	0	4.219141	-1.25927	2.449994
15	6	0	4.634407	0.012519	0.756639
16	6	0	4.115493	0.720879	-0.32675
17	1	0	4.766808	1.286661	-0.98206
18	6	0	2.74552	0.697574	-0.5579
19	6	0	1.980287	1.357056	-1.61124
20	6	0	-0.22088	1.777498	-2.58609
21	1	0	-1.30247	1.650399	-2.60123
22	6	0	0.386055	2.555434	-3.55985
23	6	0	1.758708	2.759927	-3.6002
24	1	0	2.220219	3.371671	-4.3694
25	6	0	2.526083	2.154992	-2.623
26	1	0	5.706213	0.029651	0.941407
27	6	0	-1.94804	-0.04527	-0.33576
28	6	0	-3.17928	-0.03019	-0.51007
29	6	0	-4.54684	-0.013	-0.69613
30	6	0	-5.14752	0.738046	-1.77135
31	1	0	-4.49271	1.277347	-2.45306
32	6	0	-6.50062	0.758994	-1.94481
33	1	0	-6.93869	1.311218	-2.77413

34	6	0	-7.36038	0.026235	-1.07027
35	6	0	-6.77945	-0.72872	-0.00566
36	1	0	-7.43367	-1.26805	0.676939
37	6	0	-5.42812	-0.74489	0.180733
38	1	0	-4.98881	-1.29856	1.008231
39	6	0	-9.38106	1.223654	-1.70932
40	6	0	-8.97903	2.473002	-1.22177
41	1	0	-8.19236	2.526137	-0.4711
42	6	0	-9.59609	3.626488	-1.6859
43	1	0	-9.28126	4.593576	-1.29883
44	6	0	-10.6195	3.547892	-2.62768
45	1	0	-11.1017	4.454337	-2.98758
46	6	0	-11.0238	2.302337	-3.10613
47	1	0	-11.8175	2.233052	-3.84746
48	6	0	-10.4091	1.142867	-2.65628
49	1	0	-10.7093	0.169439	-3.04001
50	6	0	-9.51159	-1.10923	-0.98304
51	6	0	-10.7411	-0.99789	-0.32306
52	1	0	-11.0946	-0.01705	-0.01025
53	6	0	-11.4872	-2.1376	-0.05999
54	1	0	-12.4396	-2.04527	0.458617
55	6	0	-11.0177	-3.39334	-0.44243
56	1	0	-11.6051	-4.28394	-0.2293
57	6	0	-9.79297	-3.5024	-1.09697
58	1	0	-9.42423	-4.4773	-1.40969
59	6	0	-9.04114	-2.36863	-1.37324
60	1	0	-8.09413	-2.44521	-1.90469
61	7	0	-8.74015	0.047441	-1.25172
62	9	0	-1.75027	-3.25098	4.079213
63	9	0	2.819492	-2.40185	3.73028
64	9	0	-0.36747	3.136728	-4.50008
65	9	0	3.851231	2.361708	-2.67541

Table S15: Optimized T_3 (IL) geometry of complex **2**.

Center	Atomic	Atomic	Coordinates (Angstroms)		
Number	Number	Type	X	Y	Z
1	79	0	-0.0131	0.017597	-0.00449
2	6	0	-0.01513	0.065776	2.083475
3	7	0	1.974177	0.003835	0.329979
4	6	0	0.63065	-0.03143	-1.9662
5	6	0	-1.08066	0.100955	2.967923
6	1	0	-2.11134	0.105336	2.617216
7	6	0	-0.82462	0.131635	4.33475
8	6	0	0.459428	0.128976	4.859062
9	1	0	0.635637	0.153047	5.929971
10	6	0	1.513965	0.094222	3.967117
11	6	0	1.324915	0.062216	2.58052
12	6	0	2.397456	0.02686	1.588779
13	6	0	3.798677	0.014916	1.836927

14	1	0	4.168034	0.034802	2.854195
15	6	0	4.693417	-0.02296	0.738457
16	6	0	4.229847	-0.0471	-0.55081
17	1	0	4.91687	-0.07567	-1.38844
18	6	0	2.81361	-0.03358	-0.80191
19	6	0	2.108836	-0.0525	-2.01221
20	6	0	-0.09718	-0.04403	-3.13561
21	1	0	-1.18592	-0.02839	-3.11514
22	6	0	0.563749	-0.07886	-4.36868
23	6	0	1.973386	-0.10279	-4.46912
24	1	0	2.460955	-0.13163	-5.43937
25	6	0	2.707687	-0.09001	-3.32151
26	1	0	5.763332	-0.03263	0.930493
27	6	0	-1.95959	0.025579	-0.33017
28	6	0	-3.16818	0.02688	-0.51301
29	6	0	-4.58	0.025926	-0.71738
30	6	0	-5.28558	1.226339	-0.8946
31	1	0	-4.73772	2.167338	-0.88967
32	6	0	-6.65668	1.225787	-1.09053
33	1	0	-7.18692	2.165116	-1.23876
34	6	0	-7.37421	0.023506	-1.09932
35	6	0	-6.67806	-1.17785	-0.91832
36	1	0	-7.22803	-2.11765	-0.91848
37	6	0	-5.30469	-1.17588	-0.73906
38	1	0	-4.77494	-2.1164	-0.59593
39	6	0	-9.57367	1.018125	-0.67781
40	6	0	-9.3152	1.433684	0.632805
41	1	0	-8.4923	0.979431	1.182525
42	6	0	-10.1008	2.416217	1.2223
43	1	0	-9.88631	2.728392	2.243291
44	6	0	-11.1636	2.985822	0.525832
45	1	0	-11.7807	3.7506	0.993462
46	6	0	-11.4279	2.566316	-0.77548
47	1	0	-12.2512	3.007911	-1.33494
48	6	0	-10.6364	1.597261	-1.37923
49	1	0	-10.836	1.279047	-2.40136
50	6	0	-9.38315	-0.98716	-2.07041
51	6	0	-10.5745	-1.58631	-1.64786
52	1	0	-11.0222	-1.27153	-0.70641
53	6	0	-11.1763	-2.56861	-2.42431
54	1	0	-12.1037	-3.02551	-2.08204
55	6	0	-10.5939	-2.9803	-3.62015
56	1	0	-11.0643	-3.75535	-4.22218
57	6	0	-9.40337	-2.3905	-4.03772
58	1	0	-8.94052	-2.69782	-4.97428
59	6	0	-8.80408	-1.39449	-3.27662
60	1	0	-7.88109	-0.92347	-3.61116
61	7	0	-8.77341	0.022119	-1.2867
62	9	0	-1.84698	0.164938	5.185248
63	9	0	2.74839	0.092534	4.485544

64	9	0	-0.14027	-0.0925	-5.50143
65	9	0	4.046423	-0.11262	-3.41404

Table S16: Optimized T_1^{cop} geometry of complex **2**.

Center	Atomic	Atomic	Coordinates (Angstroms)		
Number	Number	Type	X	Y	Z
1	79	0	-0.01466	-0.07662	0.014092
2	6	0	0.009471	-0.16933	2.089253
3	7	0	1.9552	0.013617	0.301187
4	6	0	0.594319	0.060337	-1.96772
5	6	0	-1.0566	-0.27374	2.977917
6	1	0	-2.08652	-0.3126	2.624828
7	6	0	-0.80266	-0.32913	4.338673
8	6	0	0.48503	-0.28789	4.858154
9	1	0	0.669022	-0.33438	5.927331
10	6	0	1.532651	-0.18583	3.964062
11	6	0	1.350347	-0.12156	2.577815
12	6	0	2.420079	-0.00695	1.596229
13	6	0	3.789502	0.08703	1.802559
14	1	0	4.186171	0.070543	2.811122
15	6	0	4.641142	0.204585	0.7024
16	6	0	4.136954	0.230725	-0.59963
17	1	0	4.80488	0.325818	-1.44736
18	6	0	2.767196	0.134967	-0.80348
19	6	0	2.017792	0.148762	-2.0523
20	6	0	-0.17902	0.070062	-3.12449
21	1	0	-1.26544	0.002994	-3.07963
22	6	0	0.444589	0.169358	-4.35732
23	6	0	1.824896	0.258679	-4.48732
24	1	0	2.299954	0.336402	-5.4607
25	6	0	2.580137	0.245223	-3.33088
26	1	0	5.714657	0.280749	0.861545
27	6	0	-1.95336	-0.16279	-0.2604
28	6	0	-3.18515	-0.17357	-0.42664
29	6	0	-4.55126	-0.17241	-0.61893
30	6	0	-5.46294	-0.13153	0.490638
31	1	0	-5.0502	-0.0886	1.496546
32	6	0	-6.81196	-0.11543	0.295198
33	1	0	-7.48627	-0.05156	1.146401
34	6	0	-7.35595	-0.1256	-1.0234
35	6	0	-6.46281	-0.17256	-2.13476
36	1	0	-6.87056	-0.22183	-3.14205
37	6	0	-5.11406	-0.2022	-1.9404
38	1	0	-4.43539	-0.26354	-2.78869
39	6	0	-9.60896	-0.6452	-0.26083
40	6	0	-9.34432	-1.91021	0.273582
41	1	0	-8.46989	-2.4635	-0.06477
42	6	0	-10.2086	-2.45176	1.215614
43	1	0	-10.0042	-3.43835	1.626359

44	6	0	-11.3382	-1.74492	1.621334
45	1	0	-12.0146	-2.17349	2.35791
46	6	0	-11.6031	-0.48837	1.079189
47	1	0	-12.4813	0.070105	1.396673
48	6	0	-10.7439	0.06579	0.140903
49	1	0	-10.9342	1.05258	-0.27764
50	6	0	-9.27396	0.547759	-2.36549
51	6	0	-10.3381	-0.05066	-3.04826
52	1	0	-10.7289	-1.00601	-2.70256
53	6	0	-10.8737	0.574006	-4.16544
54	1	0	-11.6963	0.102107	-4.69894
55	6	0	-10.3555	1.789119	-4.61049
56	1	0	-10.7783	2.272817	-5.48857
57	6	0	-9.29854	2.384223	-3.92598
58	1	0	-8.89844	3.339058	-4.26075
59	6	0	-8.75851	1.77281	-2.80289
60	1	0	-7.94759	2.242683	-2.249
61	7	0	-8.72384	-0.0824	-1.21891
62	9	0	-1.82753	-0.42598	5.196156
63	9	0	2.772477	-0.14992	4.479823
64	9	0	-0.29805	0.182167	-5.47218
65	9	0	3.913129	0.329156	-3.47326

Table S17: Optimized T_1^{perp} geometry of complex **2**.

Center Number	Atomic Number	Atomic Type	Coordinates (Angstroms) X	Y	Z
1	79	0	-0.00456	0.047611	0.012447
2	6	0	0.019203	-0.25417	2.076171
3	7	0	1.965492	0.052946	0.3191
4	6	0	0.647762	0.26958	-1.95555
5	6	0	-1.06219	-0.36974	2.949201
6	1	0	-2.0914	-0.33561	2.595578
7	6	0	-0.8244	-0.5391	4.302206
8	6	0	0.460723	-0.5878	4.830516
9	1	0	0.633412	-0.71343	5.895327
10	6	0	1.518792	-0.46605	3.95335
11	6	0	1.357878	-0.30125	2.57073
12	6	0	2.43211	-0.18484	1.604883
13	6	0	3.798515	-0.28926	1.801161
14	1	0	4.185075	-0.46412	2.799163
15	6	0	4.669931	-0.17605	0.711287
16	6	0	4.170332	0.030756	-0.58027
17	1	0	4.844532	0.099226	-1.42644
18	6	0	2.805322	0.136966	-0.78346
19	6	0	2.073851	0.309606	-2.02273
20	6	0	-0.11993	0.410786	-3.11111
21	1	0	-1.20798	0.383168	-3.08386
22	6	0	0.515294	0.587313	-4.3279
23	6	0	1.899985	0.642032	-4.44191

24	1	0	2.385303	0.789663	-5.40214
25	6	0	2.644461	0.504613	-3.28822
26	1	0	5.742883	-0.26526	0.866568
27	6	0	-1.94241	0.10822	-0.27296
28	6	0	-3.16938	0.102474	-0.45581
29	6	0	-4.54529	0.088421	-0.6556
30	6	0	-5.2767	1.296912	-0.81949
31	1	0	-4.73247	2.238549	-0.80963
32	6	0	-6.63235	1.283607	-1.00846
33	1	0	-7.17059	2.215818	-1.1594
34	6	0	-7.33966	0.053269	-1.05641
35	6	0	-6.61756	-1.15906	-0.89698
36	1	0	-7.15281	-2.10513	-0.89905
37	6	0	-5.26365	-1.13825	-0.69843
38	1	0	-4.71633	-2.0666	-0.55132
39	6	0	-9.5125	1.161353	-0.93171
40	6	0	-9.40978	1.761265	0.325505
41	1	0	-8.7025	1.368927	1.054664
42	6	0	-10.2255	2.842179	0.635396
43	1	0	-10.1486	3.307304	1.615862
44	6	0	-11.1451	3.316958	-0.29632
45	1	0	-11.7837	4.161934	-0.04765
46	6	0	-11.2496	2.707699	-1.54506
47	1	0	-11.9644	3.079177	-2.27628
48	6	0	-10.4372	1.629259	-1.86734
49	1	0	-10.5061	1.14849	-2.84173
50	6	0	-9.35233	-1.1082	-1.8031
51	6	0	-10.5008	-1.60396	-1.18309
52	1	0	-10.8675	-1.12931	-0.27453
53	6	0	-11.1515	-2.70212	-1.72882
54	1	0	-12.0418	-3.09503	-1.24247
55	6	0	-10.6644	-3.30257	-2.88814
56	1	0	-11.1784	-4.16212	-3.31305
57	6	0	-9.5228	-2.79887	-3.50654
58	1	0	-9.14685	-3.25599	-4.41936
59	6	0	-8.8656	-1.69831	-2.97164
60	1	0	-7.98408	-1.28365	-3.45824
61	7	0	-8.69875	0.035406	-1.25566
62	9	0	-1.86267	-0.66394	5.143829
63	9	0	2.754707	-0.50833	4.480482
64	9	0	-0.21961	0.71131	-5.44441
65	9	0	3.981322	0.568803	-3.4141

References

[1] A. J. Wilkinson, H. Puschmann, J. A. K. Howard, C. E. Foster, J. A. G. Williams, *Inorg. Chem.* **2006**, *45*, 8685–8699.

[2] a) K.-H. Wong, K.-K. Cheung, M. C.-W. Chan, C.-M. Che, *Organometallics* **1998**, *17*, 3505–3511; b) J. Fernandez-Cestau, B. Bertrand, M. Blaya, G. A. Jones, T. J. Penfold, M. Bochmann, *Chem. Commun.* **2015**, *51*, 16629–16632.

[3] A. Ito, K. Kawanishi, E. Sakuda, N. Kitamura, *Chem. Eur. J.* **2014**, *20*, 3940–3953.

[4] Y.-D. Lin, C.-T. Chien, S.-Y. Lin, H.-H. Chang, C.-Y. Liu, T. J. Chow, *J. Photochem. Photobiol. A: Chem.* **2011**, *222*, 192–202.

[5] G. Xie, X. Li, D. Chen, Z. Wang, X. Cai, D. Chen, Y. Li, K. Liu, Y. Cao, S.-J. Su, *Adv. Mater.* **2016**, *28*, 181–187.

[6] Y. Kitamoto, T. Namikawa, D. Ikemizu, Y. Miyata, T. Suzuki, H. Kita, T. Sato, S. Oi, *J. Mater. Chem. C.* **2015**, *3*, 9122–9130.

[7] H. Guo, M. L. Muro-Small, S. Ji, J. Zhao, F. N. Castellano, *Inorg. Chem.* **2010**, *49*, 6802–6804.

[8] a) C. Féry, B. Racine, D. Vaufrey, H. Doyeux, S. Cinà, *Appl. Phys. Lett.* **2005**, *87*, 213502; b) T. Fleetham, Y. Ji, L. Huang, T. S. Fleetham, J. Li, *Chem. Sci.* **2017**, *8*, 7983–7990.

[9] L.-K. Li, M.-C. Tang, S.-L. Lai, M. Ng, W.-K. Kwok, M.-Y. Chan, V. W.-W. Yam, *Nat. Photonics* **2019**, *13*, 185–191.

[10] Y. Zhao, D. G. Truhlar, *Theor. Chim. Acta* **2008**, *120*, 215–241.

[11] M. J. Frisch, G. W. Trucks, H. B. Schlegel, G. E. Scuseria, M. A. Robb, J. R. Cheeseman, G. Scalmani, V. Barone, B. Mennucci, G. A. Petersson, H. Nakatsuji, M. Caricato, X. Li, H. P. Hratchian, A. F. Izmaylov, J. Bloino, G. Zheng, J. L. Sonnenberg, M. Hada, M. Ehara, K. Toyota, R. Fukuda, J. Hasegawa, M. Ishida, T. Nakajima, Y. Honda, O. Kitao, H. Nakai, T. Vreven, J. A. Montgomery Jr, J. E. Peralta, F. Ogliaro, M. Bearpark, J. J. Heyd, E. Brothers, K. N. Kudin, V. N. Staroverov, R. Kobayashi, J. Normand, K. Raghavachari, A. Rendell, J. C. Burant, S. S. Iyengar, J. Tomasi, M. Cossi, N. Rega, J. M. Millam, M. Klene, J. E. Knox, J. B. Cross, V. Bakken, C. Adamo, J. Jaramillo, R. Gomperts, R. E. Stratmann, O. Yazyev, A. J. Austin, R. Cammi, C. Pomelli, J. W. Ochterski, R. L. Martin, K. Morokuma, V. G. Zakrzewski, G. A. Voth, P. Salvador, J. J. Dannenberg, S. Dapprich, A. D. Daniels, Ö. Farkas, J. B. Foresman, J. V. Ortiz, J. Cioslowski, D. J. Fox, Revision D.01 ed., Gaussian, Inc., Wallingford CT, **2009**.

[12] a) M. M. Francl, W. J. Petro, W. J. Hehre, J. S. Binkley, M. S. Gordon, D. J. DeFree, J. A. Pople, *J. Chem. Phys.* **1982**, *77*, 3654-3665; b) P. C. Hariharan, J. A. Pople, *Theor. Chim. Acta* **1973**, *28*, 213-222.

[13] a) D. Andrae, U. Haeussermann, M. Dolg, H. Stoll, H. Preuss, *Theor. Chim. Acta* **1990**, *77*, 123-141; b) J. M. L. Martin, A. Sundermann, *J. Chem. Phys.* **2001**, *114*, 3408-3420.

[14] M. Cossi, G. Scalmani, N. Rega, V. Barone, *J. Chem. Phys.* **2002**, *117*, 43-54.

[15] a) R. Improta, V. Barone, G. Scalmani, M. J. Frisch, *J. Chem. Phys.* **2006**, *125*, 054103; b) R. Improta, G. Scalmani, M. J. Frisch, V. Barone, *J. Chem. Phys.* **2007**, *127*, 074504.

[16] a) G. S. M. Tong, C.-M. Che, *Chem. Eur. J.* **2009**, *15*, 7225-7237; b) G. S. M. Tong, P. K. Chow, W.-P. To, W.-M. Kwok, C.-M. Che, *Chem. Eur. J.* **2014**, *20*, 6433-6443; c) G. S. M. Tong, K. T. Chan, X. Chang, C.-M. Che, *Chem. Sci.* **2015**, *6*, 3026-3037.

[17] S. Hirata, M. Head-Gordon, *Chem. Phys. Lett.* **1999**, *314*, 291-299.

[18] a) M. E. Casida, F. Gutierrez, J. Guan, F.-X. Gadea, d. Salahub, J.-P. Daudey, *J. Chem. Phys.* **2000**, *113*, 7062-7071; b) M. J. G. Peach, M. J. Williamson, D. J. Tozer, J. Chem. *Theory Comput.* **2011**, *7*, 3578-3585.

[19] a) R. Czerwieniec, M. J. Leitl, H. H. H. Homeier, H. Yersin, *Coord. Chem. Rev.* **2016**, *325*, 2-28; b) M. Z. Shafikov, A. F. Suleymanova, R. Czerwieniec, H. Yersin, *Chem. Mater.* **2017**, *29*, 1708-1715; c) W.-P. To, D. Zhou, G. S. M. Tong, G. Cheng, C. Yang, C.-M. Che, *Angew. Chem. Int. Ed.* **2017**, *56*, 14036-14041; *Angew. Chem.* **2017**, *129*, 14224-14229.

[20] T. Lu, F. Chen, *J. Comput. Chem.* **2012**, *33*, 580-592.
